# Number Sense and Mathematics: Which, When and How?

**DOI:** 10.1037/dev0000331

**Published:** 2017-07-31

**Authors:** Maria G. Tosto, Stephen A. Petrill, Sergey Malykh, Karim Malki, Claire M. A. Haworth, Michele M. M. Mazzocco, Lee Thompson, John Opfer, Olga Y. Bogdanova, Yulia Kovas

**Affiliations:** 1 Department of Psychology, Tomsk State University; 2 Department of Psychology, The Ohio State University; 3 Psychological Institute of RAE, Moscow, Russia; 4 King’s College London at the Institute of Psychiatry, Psychology and Neuroscience (IOPPN); 5 School of Experimental Psychology and The Medical Research Council Integrative Epidemiology Unit, School of Social and Community Medicine, University of Bristol; 6 Institute of Child Development, University of Minnesota; 7 Department of Psychology, The Ohio State University; 8 Department of Psychology, Tomsk State University; 9 Department of Psychology, Tomsk State University, and Department of Psychology, Goldsmiths, University of London

**Keywords:** mathematics, number sense, symbolic estimation, nonsymbolic estimation

## Abstract

Individual differences in number sense correlate with mathematical ability and performance, although the presence and strength of this relationship differs across studies. Inconsistencies in the literature may stem from heterogeneity of number sense and mathematical ability constructs. Sample characteristics may also play a role as changes in the relationship between number sense and mathematics may differ across development and cultural contexts. In this study, 4,984 16-year-old students were assessed on estimation ability, one aspect of number sense. Estimation was measured using 2 different tasks: number line and dot-comparison. Using cognitive and achievement data previously collected from these students at ages 7, 9, 10, 12, and 14, the study explored for *which* of the measures and *when* in development these links are observed, and *how* strong these links are and *how* much these links are moderated by other cognitive abilities. The 2 number sense measures correlated modestly with each other (*r* = .22), but moderately with mathematics at age 16. Both measures were also associated with earlier mathematics; but this association was uneven across development and was moderated by other cognitive abilities.

“Number sense” is a term used to describe a wide range of mathematically relevant concepts, with up to 30 different constructs falling under this broad definition (e.g., Berch, 2005). Estimation, one aspect of number sense, is associated with quantifying and representing number magnitudes and numerosities (discrete items in a set). Estimation is itself heterogeneous, involving different abilities, such as nonsymbolic estimation and symbolic estimation (see Cohen Kadosh, Lammertyn, & Izard, 2008). These skills have been associated with mathematics, although questions remain about the extent to which this association varies depending on specific estimation tasks and periods of development.

## Nonsymbolic Estimation and Its Relationship With Mathematics

Nonsymbolic estimation involves nonverbal processing of quantities and numerosities without using numerals. For example, this ability enables us to select a queue with fewer people without counting. Research suggests that this type of numerosity processing depends on the absolute number of items in a set: Evaluation of individual sets including fewer items is more accurate compared with those containing more items (set-size effect; e.g., Gordon, 2004; Whalen, Gallistel, & Gelman, 1999). Furthermore, discrimination between two sets is more difficult when the discrepancy between the number of items in the sets is smaller (distance effect; e.g., Feigenson, Carey, & Hauser, 2002; Holloway & Ansari, 2009; Moyer & Landauer, 1967). These two effects are encompassed by Weber’s law, with the Weber Fraction indexing the minimum ratio between two sets reliably discernible by individuals (Weber, 1834).

Numerosity processing can be carried out without formal knowledge of numbers or formal instruction (e.g., Pica, Lemer, Izard, & Dehaene, 2004) and, in humans, this skill improves with development. For example, 6-month-old babies can successfully discriminate only between large ratios, such as 8 versus 16 (ratios 1:2), with corresponding Weber Fraction of 1 ([(2 − 1)÷1]; e.g., Libertus & Brannon, 2010). Adults can discriminate larger numerosities and smaller ratios (Halberda & Feigenson, 2008; Halberda, Ly, Wilmer, Naiman, & Germine, 2012).

People differ greatly in the speed and accuracy of estimation (e.g., Halberda, Mazzocco, & Feigenson, 2008). Individual differences in nonsymbolic estimation, assessed using different nonsymbolic tasks, have been found in preschoolers, school-age children, and adults (e.g., Barth et al., 2006; Gilmore, McCarthy, & Spelke, 2010; Halberda et al., 2012; Nys & Content, 2012). A few studies that looked at potential sex differences in nonsymbolic estimation found no average differences between males and females (e.g., 3–5-year-olds, Bonny & Lourenco, 2013; 5–6-year-olds, Gilmore et al., 2010; 4-year-olds, Libertus, Feigenson, & Halberda, 2011; 14–15-year-olds, Mazzocco, Feigenson, & Halberda, 2011a). However, one study reported a small male advantage in 4-year-olds (Soltész, Szücs, & Szücs, 2010).

Several longitudinal studies showed an association between individual differences in nonsymbolic estimation and mathematical performance in preschool children (Gilmore et al., 2010; Mazzocco, Feigenson, & Halberda, 2011b) and older children (Halberda & Feigenson, 2008), with evidence suggesting a causal association (Wang, Odic, Halberda, & Feigenson, 2016). However, other studies have failed to find a significant correlation (e.g., Holloway & Ansari, 2009; Rousselle & Noël, 2007; Sasanguie, Defever, Maertens, & Reynvoet, 2014).

Despite inconsistencies across individual studies, meta-analyses have shown that nonsymbolic estimation is prospectively and retrospectively, weakly, associated with mathematics across development (*r* = .24 prospectively and .17 retrospectively, Chen & Li, 2014; *r* = .22, Fazio, Bailey, Thompson, & Siegler, 2014; *r* = .24, Schneider et al., 2017). Discrepancies across individual studies may have stemmed from: differences in age of participants (Fazio et al., 2014; Schneider et al., 2017); measures of estimation used (for a discussion see Clayton, Gilmore, & Inglis, 2015); specific mathematics skills with which estimation is being correlated (Mazzocco et al., 2011a); mathematics achievement level of the participants (Bonny & Lourenco, 2013; Mazzocco et al., 2011a); and overall lack of statistical power to detect weak associations.

## Symbolic Estimation and Its Relationship With Mathematics

Symbolic estimation relies on symbols, such as Arabic numerals (Booth & Siegler, 2006; Cohen Kadosh, et al., 2008). For example, by relying on symbolic estimation people can tell that the solution to a numerical problem is incorrect without calculating an exact answer. The size and ratio effects observed for nonsymbolic estimation are also observed for symbolic estimation. Overall, people are faster in comparing two small numbers (1 and 2) than two large numbers (8 and 9) even when the distance between them is kept constant, suggesting that it is easier to process small numbers (Moyer & Landauer, 1967). Moreover, adults and children are faster and more accurate in judging the difference between two numerical magnitudes when the numerical distance between the numerals is larger (1 vs. 9) than when it is smaller (6 vs. 8; e.g., Dehaene, Dupoux, & Mehler, 1990). The presence of size and ratio effects in symbolic estimation has been taken as indirect evidence that symbolic representation of numbers builds on the approximate representation of nonsymbolic numerosity (Feigenson, Dehaene, & Spelke, 2004). The closeness between symbolic and nonsymbolic estimation seems also supported by reliance on partially overlapping neuronal activity in the intraparietal sulcus (IPS) and prefrontal cortex (for a discussion see Nieder & Dehaene, 2009). IPS areas are activated when attending to numerosity stimuli (e.g., Piazza, Izard, Pinel, Le Bihan, & Dehaene, 2004) or manipulating Arabic number symbols (e.g., Pinel, Dehaene, Riviere, & LeBihan, 2001). Different neurons in parietal regions, respond to a specific numerosity (tuning function); such tuning functions are organized sequentially, preserving the order of cardinality (numerosity of a set size) and following the Weber law (Nieder & Merten, 2007). However, some neural pathways show differential activation during encoding of numerical magnitudes gathered from symbolic and nonsymbolic stimuli (Holloway, Price, & Ansari, 2010). Furthermore, there is evidence of lateralization in IPS response to symbolic and nonsymbolic processing (Holloway, Battista, Vogel, & Ansari, 2013).

It is thought that, as numerals are acquired, they map onto existing nonsymbolic representations and become mentally represented along a mental “number line” (e.g., Restle, 1970; Siegler & Opfer, 2003). This line is organized in ascending order, following a left-to-right direction in English-writing participants and right-to-left in Arabic-writing participants (Dehaene, Bossini, & Giraux, 1993; cf. Ito & Hatta, 2004). It is hypothesized that numbers on the mental number line are initially logarithmically compressed (e.g., Dehaene & Mehler, 1992). With age, a gradual shift seems to occur from the less accurate logarithmic mental number representation to a more precise linear representation. The linear representation becomes dominant from the age of 6 to 8 years, as evidenced by improved performance on the number line task (Siegler & Booth, 2004). However, performance on this task may be based on strategies such as reliance on midpoint (knowing that 50 is half of 100; Ashcraft & Moore, 2012) and reliance on proportion-judgment, as the position of a number on a number line is estimated relatively to the size of the whole line (Barth & Paladino, 2011). Therefore, developmental changes may be due to the increasing use of a reference point rather than a log-to-linear shift. Another explanation for the increased accuracy on number line tasks takes into account familiarity with number symbols (e.g., Ebersbach, Luwel, Frick, Onghena, & Verschaffel, 2008; Moeller, Pixner, Kaufmann, & Nuerk, 2009). These explanations are not mutually exclusive (Dackermann, Huber, Bahnmueller, Nuerk, & Moeller, 2015).

Several studies in different cultures have found a correlation between performance on number line tasks and mathematics skills (e.g., Booth & Siegler, 2006; Fazio et al., 2014; Fuchs et al., 2010a; Geary, 2011; Siegler & Booth, 2004; Siegler & Mu, 2008). The mechanisms of the association are unclear. Research suggests that experience with numbers, such as playing numerical board games, can improve children’s estimation abilities on the number line (Siegler & Booth, 2004). In turn, improvement of magnitude processing on the number line was found to be causally related to better arithmetic (addition problems) skills (Booth & Siegler, 2008). However bidirectional effects are also likely. For example, it was found that access to numerical instruction can improve nonsymbolic estimation skills in Western adults (Nys et al., 2013). In children, the association between nonsymbolic estimation and mathematics was found to be mediated by symbolic estimation skills, such as knowledge of number words and Arabic numerals and of their meaning (cf. Räsänen, Salminen, Wilson, Aunio, & Dehaene, 2009; van Marle, Chu, Li, & Geary, 2014). It is possible that number line activities contribute to the knowledge of symbolic quantities, which is one of the most powerful predictors of later achievement (Duncan et al., 2007; Jordan, Kaplan, Ramineni, & Locuniak, 2009).

Similar to nonsymbolic estimation, there is evidence pointing to a small male advantage in number line estimation (Hannula, 2003; LeFevre et al., 2010), although these results are not consistent (Gunderson, Ramirez, Beilock, & Levine, 2012; Thompson & Opfer, 2008).

## Nonsymbolic and Symbolic Estimation and Other Cognitive Abilities

A wealth of previous research has found associations between mathematics and other non-numerical abilities, such as working memory (e.g., Bull, Johnston, & Roy, 1999; Geary, 2011; McLean & Hitch, 1999; Siegel & Ryan, 1989; Swanson & Sachse-Lee, 2001); speed of processing (Bull & Johnston, 1997; Bull et al., 1999; Case, Kurland, & Goldberg, 1982); and reading and general cognitive factors (e.g., Dirks, Spyer, van Lieshout, & de Sonneville, 2008; Fuchs et al., 2010b; Kovas, Harlaar, Petrill, & Plomin, 2005; Kovas, Haworth, Petrill, & Plomin, 2007). Less is known about the role of these abilities in the link between mathematics and nonsymbolic and symbolic estimation.

One study found that the correlation between a nonsymbolic (dot) discrimination task at age 14 and mathematical ability at age 8 remained significant after controlling for 16 cognitive measures assessed at age 8, including visuospatial reasoning, working memory, reading, word knowledge, and object perception (Halberda & Feigenson, 2008). Similarly, nonsymbolic estimation skills were significantly correlated with mathematics in over 10,000 11-to 85-year-old participants, after controlling for age, sex, as well as measures of science, writing and computer ability (Halberda et al., 2012). In preschoolers nonsymbolic estimation skills were associated with mathematical abilities, but not with vocabulary or letter identification in early primary school (Mazzocco et al., 2011b). However, another study found that a nonsymbolic (dot) discrimination task correlated only with short-term memory but not with counting and number knowledge in 4–7-year-olds (Soltész et al., 2010). Number line estimation has been linked to individual differences in IQ and in aspects of working memory in 7–8-year-old children (Geary, Hoard, Nugent, & Byrd-Craven, 2008) and with visuospatial skills (Bachot, Gevers, Fias, & Roeyers, 2005).

## The Present Study

The body of knowledge on the links between estimation, other cognitive abilities, and mathematics is growing. However, most of the studies into symbolic and nonsymbolic estimation have been conducted in early to middle childhood. Furthermore, most studies have used only a few measures, and therefore meta-analyses draw conclusions based on widely differing measures and ages (see Schneider et al., 2017). Previous research provided inconsistent findings regarding the presence of sex differences in estimation abilities. It is therefore unclear whether sex differences in estimation, if found, may contribute to the observed sex differences in mathematical ability (e.g., Spelke, 2005).

The present study is a large-scale multivariate investigation into the relationship between two aspects of number sense and formal mathematics across development. The study has three major aims: (1) to examine the relationship between nonsymbolic and symbolic estimation abilities, as assessed by a dot estimation and a number line tasks at age 16; (2) to assess whether estimation abilities measured at age 16 are related with mathematical abilities measured at ages 7, 9, 10, 12, 14, and 16; (3) to assess whether the links between mathematical ability and estimation are present after accounting for a number of verbal and non-verbal abilities measured in the same children at 7, 9, 10, 12, 14, and 16 years of age. The large sample used in the study affords a statistically powerful evaluation of potential sex differences in estimation and in the extent to which sex differences in estimation are associated with sex differences in mathematical ability.

## Method

### Participants

Participants were drawn from the longitudinal, U.K. representative Twins Early Development Study (TEDS) sample (Haworth, Davis, & Plomin, 2013). Families of twins born between 1994 and 1996 in England and Wales were identified through birth records. Out of the 16,810 families recruited into the study, over 12,000 remain active. The project received approval from the King’s College London Institute of Psychiatry ethics committee. For each assessment, informed consent was obtained from parents before data collection and the twins gave their assent.

The current report is based on cognitive abilities and school achievement data collected when the twins were 7 (*M*_age_ = 7.12, *SD* = .25), 9 (*M*_age_ = 9.03, *SD* = .28), 10 (*M*_age_ = 10.09, *SD* = .28), 12 (*M*_age_ = 11.65, *SD* = .68), 14 (*M*_age_ = 14.08, *SD* = .57), and 16 (*M*_age_ = 16.58, *SD* = .30) years old. Data were excluded from twins for whom English is not their first language and those with severe medical conditions, psychiatric disorders, and perinatal complications. These criteria generated a sample of 17,882 individuals (9,175 females, from 8,941 families) who contributed at least one data point.

Not all twins were tested at each assessment wave (see supplementary online materials **[**SOM**]** for further details). This led to only partially overlapping samples across ages; therefore, homogeneity and representativeness of the samples over time were assessed in order to ensure meaningful comparisons across ages. First, quantile regressions assessed (1) whether the strength of associations was similar across the 25^th^, 50^th^, and 75^th^ quantiles of each measure (details in SOM and Figures S5 and S6); and (2) the stability of the associations across development. These analyses showed very similar patterns across the quantiles (homogeneity), justifying the use of mean analyses. Furthermore, the associations were stable across ages, showing very similar results in the partially overlapping samples.

Second, we compared socioeconomic status (SES), assessed when the twins were about one and a half years old, across the partially overlapping groups at ages of 7, 9, 10, 12, 14, and 16 years. These analyses (detailed in Table S1, SOM) showed significant but very small mean SES differences between ages 7 and 12, 7 and 14, and 7 and 16, with effect size ranging between .07 and .12 when computed in r, and between .10 and .24 in Cohen’s d. In the comparison of all groups, the effect size, computed in r, ranged between .03 and .12, and in Cohen’s d ranged between .07 and .24, suggesting little effects of the missing data. Furthermore, the TEDS sample has shown to be representative of the same age U.K. population over the years (Haworth et al., 2013). Overall, these analyses suggest that it is unlikely that the results are affected by the different composition of the samples. Given the diverse causes of unavailability and the little effects of the missing data, no imputation was conducted and missing data was treated using listwise deletion.

Analyses were conducted on data from one randomly selected twin in each pair and replicated on the second half of the sample. Only results statistically significant in both samples were considered significant. This stringent approach ensures independence of data and guards against chance or practically insignificant findings.

### Measures

#### Measures age 16

Data measuring symbolic and nonsymbolic estimation, mathematics, and a range of cognitive abilities were collected using 11 computerized tests administered online, briefly described below and summarized in Table 1. More details about these tests and recruitment of the sample at age 16 can be found in SOM. The age of 16 corresponds to the end of the compulsory education in the U.K., and students take a public examination (GCSE: General Certificate of Secondary Education). We used the mathematics GCSE scores as a further measure of mathematical ability at this age.[Table-anchor tbl1]

**Estimation Ability** was measured with two tasks. The *Dot Task,* adapted from Halberda and Feigenson (2008), is used to assess nonsymbolic approximate estimation of large numerosities. The task consists of 150 trials depicting arrays with interspersed yellow and blue dots. These stimuli remain on the screen for 400 ms, during which time the participant selects whether the display contains more yellow or blue dots, by pressing “Y” for more yellow and “B” for more blue dots. A Weber Fraction score was derived as a measure of the numerical ratio at which a participant’s numerical discrimination is reliably accurate, which in turn indicates the precision of numerical estimation (details in SOM). Weber Fraction scores correlated over 98% with accuracy (proportion of correct answers) on this task; analyses conducted using both accuracy and Weber Fraction scores yielded very similar results. Here we report only results on the Weber Fraction scores and refer to them as “dot estimation.” The *Number Line* task, adapted from Opfer and Siegler (2007), assesses understanding of numerical magnitudes and ability to estimate the size of numbers. A line, with the left edge marked with “0” and the right edge marked with “1000,” is presented with a numeral above it. Participants indicate the position of numerals (22 in this test) by dragging and releasing a cursor along the line, using a computer mouse. The numbers on the number line are programmed as deviations in pixels from “0”; participants’ scores represent the mean of deviations in pixels from the correct position of each number on the line. The scores were normalized with a log-10 transformation prior to the analyses. Scores on this task are referred to as “number line estimation.”

**Mathematical Performance** was measured with two Web tests and one postal questionnaire. The *Problem Verification Task,* adapted from Murphy and Mazzocco (2008), assesses calculation fluency—the efficiency with which the veracity of an arithmetic solution is evaluated and basic facts of arithmetic are retrieved. The test consists of 48 arithmetic problems such as 28 ÷ 16 = 2. Participants are asked to quickly indicate, by key-press, whether the answer is correct. Number of correct answers was used for the analyses. *Understanding Numbers* measures mathematical skills according to the achievement level required by the U.K. National Curriculum at age 16 (e.g., Tosto, Asbury, Mazzocco, Petrill, & Kovas, 2016). Items are 18 problems selected from the National Foundation for Educational Research (NFER) booklets (levels 1 to 8; nferNelson, 1994, 1999, 2001). For some questions such as “Work out the value of *x*: 6*x* + 9 = 8*x,*” response is given by clicking on the correct solution from five choices. For some problems the answer needs to be typed in. Number of correct answers was used in the analyses. The two mathematics Web tests correlated .70 and were combined together in a single score, *Mathematics web*, by averaging their standardized means. *Mathematics GCSE* scores were collected by questionnaires sent to the families, soon after the release of school examination results. Mathematics GCSE is graded from G (lowest) to A* (A-star, the highest). These grades were coded on an 8-point scale, from 4 to 11, respectively.

**General Cognitive Ability** was assessed with four tests. *Corsi Tapping Block*, adapted from Farrell Pagulayan, Busc, Medina, Bartok, and Krikorian (2006), measures visuospatial working memory. Stimuli consist of 9 small cubes arranged inside a black square. The cubes glow one at a time in a sequential pattern. Participants are asked to reproduce the pattern by clicking on the cubes with a mouse. Number of correct responses was used in the analyses. *Reaction Time*, adapted from Deary, Der, and Ford (2001), assesses speed of processing as measured by response reaction time (RT). Participants are asked to complete 40 trials in a fixed order by pressing 1, 2, 3, and 4 on the keyboard as soon as one of these numbers is presented on the screen. Prior to analyses, to account for speed-accuracy trade-off, efficiency scores were derived by dividing the median RT of correct responses by the proportion of correct answers. Efficiency scores were then normalized with a log-10 transformation. *Raven’s Progressive Matrices*, adapted from Raven, Court, and Raven (1996), assesses non-verbal (fluid) intelligence. Participants are administered a maximum of 30 trials where they complete a matrix by clicking on the missing pattern among the choice of 8. Number of correct responses was used in the analyses. *Mill Hill Vocabulary*, adapted from Raven, Raven, and Court (1998), assesses verbal ability. Participants complete 33 trials, selecting which of 6 words is similar in meaning to the target word presented on the screen. Number of correct answers was used in the analyses.

**Language Ability** was measured with the semantics *Figurative Language* subtest adapted from the Test of Language Competence (Wiig, Secord, & Sabers, 1989). The test assesses the interpretation of metaphors or figures of speech and the understanding of such nonliteral language. The stimuli consist of 15 figurative expressions referring to a situation presented in oral and written format (e.g., A boy talking about his girlfriend says, “She is easily crushed”). Participants select a matching expression from a choice of 4 (such as the following: Her bones break quite easily; She must be handled with care; She can handle anything; She has a crush on me) by clicking on it with a mouse. Number of correct responses was used in the analyses.

**Reading Ability** was measured with two tests (*r* = .4), combined into a reading composite by averaging their standardized means. The *Reading Fluency* test, adapted from Woodcock-Johnson III (Woodcock, McGrew, & Mather, 2001), consists of 98 questions requiring yes/no answers. Participants have 2 minutes and 30 seconds to answer as many questions as possible by clicking with a mouse on the “Yes” or “No” buttons appearing on the screen together with the question. Number of correct responses was used in the analyses. *Reading comprehension* test, developed by Hayiou-Thomas & Dale (available from the authors) is based on two passages of written text. Participants read the passages and answer 13 multiple choice questions for each passage. Number of correct responses was used in the analyses.

#### Validation

Prior to the main data collection, the tasks were piloted and tested for reliability and suitability for Web administration using samples of 16-years-old singleton and twin students. All tests proved to be suitable for Web administration (see SOM for details) and showed good internal consistency and test–retest reliability (see Table 2).[Table-anchor tbl2]

#### Measures age 7 to 14

Measures used at the ages 7, 9, 10, 12, and 14 are briefly listed below and summarized in Table 1. More details are presented in SOM. Detailed descriptions of the tests at these ages and their validation can be found elsewhere (e.g., Haworth et al., 2007; Kovas et al., 2007).

##### 7 years

Data for cognitive abilities (Verbal Ability, Non-Verbal Ability, and Reading) were collected using telephone testing. Mathematics school achievement was collected using teacher questionnaires.

##### 9 years

Data for cognitive abilities (Verbal Ability and Non-Verbal Ability) were collected using child-completed postal booklets. Mathematics school achievement was collected using teacher questionnaires.

##### 10 years

Data for cognitive abilities (Verbal Ability, Non-Verbal Ability, Mathematics Web and Reading) were collected using an online test battery. Mathematics school achievement was collected using teacher questionnaires.

##### 12 years

Data for cognitive abilities (Verbal Ability, Non-Verbal Ability, Mathematics Web, Spatial Ability, Language and Reading) were collected using a Web-based test battery. Mathematics school achievement was collected using teacher questionnaires.

##### 14 years

Data for cognitive abilities (Verbal Ability and Non-Verbal Ability) were collected using a Web-based test battery. Mathematics school achievement was collected using teacher questionnaires.

## Results

All measures were corrected for age and standardized to a mean of .00 and a standard deviation of 1.00; scores ± 3 standard deviations (SDs) were excluded. Descriptive statistics for the whole sample and for males and females separately are presented in Table 3 for measures at age 16, and in SOM Table S3, for measures at ages 7–14.[Table-anchor tbl3]

All tables present the results for one half of the sample. Results from the replication sample are available from the authors. As expected, the two samples were nearly identical in terms of means and distributions for all variables. The symbol ♦ indicates results that were statistically significantly different between the two samples, suggesting weak/unreliable effects.

Number line estimation and dot estimation correlated with each other modestly, *r* = .22, 95% CI [18; .26] (Table S4, SOM); we further explored their association by entering them into an exploratory factor analysis together with all the cognitive abilities measured at age 16 (Table S2, SOM). The method of the eigenvalues greater than one, suggested the extraction of two factors; however, because the initial extraction identified a third factor with an eigenvalue of .91 and the scree plot allowed the extraction of a third factor, we conducted analyses extracting two and three factors. In a two-factors model number line and dot estimation clustered together, with a three-factors model they loaded in two distinct factors (details of the analysis in SOM). The modest correlation and the results of the factor analysis suggest heterogeneity within the estimation domain, at least when assessed with a dot estimation and a number line task at age 16.

Robust correlations among cognitive abilities and achievement were observed over time (see Table S4, SOM). Smaller scores for number line estimation, dot estimation, and speed of processing index better performance, therefore correlations of these three measures are positive among each other and negative with all other measures. On average, scores from both estimation measures correlated substantially with mathematics at all ages (average *r* = −.34 and −.23 for number line and dot estimation, respectively). The two estimation measures were also significantly associated with cognitive abilities measured concurrently (age 16) and retrospectively. Average correlations were as follows: for verbal ability, *r* = −.20 (with number line) and *r* = −.15 (with dot estimation); for non-verbal ability, *r* = −.25 (with number line) and *r* = −.20 (with dot estimation); for reading, *r* = −.24 (with number line) and *r* = −.17 (with dot estimation); for language, *r* = −.24 (with number line) and *r* = −.20 (with dot estimation). On average, number line estimation yielded higher correlations with all abilities than dot estimation.

Mathematics achievement reported by teacher, mathematics Web scores, and GCSE scores showed moderate to substantial correlations with each other (*r* between .44 and .75; Table S4, SOM).

### Estimation and Mathematics Over Time

Although we had longitudinal measures of mathematical ability from age 7 to 16, estimation was only measured at age 16. To address the second aim of the study we examined the retrodictive predictions from estimation to mathematics at each age in separate regressions, entering number line and dot estimation as statistical predictors and mathematics scores as criterion variables. The results, presented in Table 4, show that symbolic and nonsymbolic estimation were significantly associated with mathematics, concurrently and retrospectively. Overall, number line estimation was more strongly associated with mathematics than dot estimation (average β = −.29 and −.16 for number line and dot estimation, respectively). The association between number line and the mathematics variables (as indexed by beta-coefficients) was overall very similar across ages. The only significant differences were found between the strength of the association at age 16 and the strength of association shown at age 7,10 and 12 (Figure S1 in SOM). A similar pattern was observed for the associations between dot estimation and mathematics over time. However, for dot estimation the differences were significant only between Teacher assessed mathematics at 14 and Mathematics web at 16 (Figure S2 in SOM). These results suggest that the association between mathematics and estimation abilities changes with the changes in mathematics phenotype; they also suggest that the association between them strengthens over time, potentially due to reciprocal influences.[Table-anchor tbl4]

Next, we explored whether early mathematical ability and achievement explained additional variance in individual differences in estimation abilities at 16, beyond concurrent mathematics. As evidence suggests that there is a strong relationship between early mathematics achievement and early number knowledge (see Geary, Hoard, Nugent, & Bailey, 2013), we used the earliest measure of mathematics in our sample to test whether it was related to estimation skills at age 16. These analyses were conducted on over 1,400 participants with complete data. Number line estimation and dot estimation were entered as criterion variables in separate stepwise regressions, mathematics at age 16 (GCSE and Web) was entered in the first step, and mathematics at age 7 was added in the second. For number line estimation, both measures of mathematics at 16 and mathematics at 7 were significant predictors with betas on the second step as follows: β = −.10, *t* = −2.68, *p* < .01 for GCSE age 16, β = −.30, *t* = −8.71, *p* < .001 for Web assessed mathematics at age 16 and β = −.09, *t* = −3.31, *p* < .01 for teacher assessed mathematics at age 7. Both mathematics measures at 16 were significant predictors of dot estimation in the first step. In the second step mathematics Web assessed at age 16 (but not GCSEs) was a significant predictor of dot estimation (β = −.20, *t* = −5.24, *p* < .001, second step) together with mathematics at age 7 (β = −.10, *t* = −3.46, *p* < .01, second step). Overall, these results suggest lasting links between late estimation and early mathematics. As estimation was available only at age 16, it is unclear whether this would also be true for early dot and number line estimation. An inspection of the 95% CI of the beta coefficients derived from these analyses (Figures S3 and S4 in SOM) suggests that teacher assessed mathematics at age 7 and exam assessed mathematics at age 16 have similar association with estimation (both measures) at age 16; furthermore, these associations were significantly different from the association between Web assessed mathematics at age 16 and estimation at age 16.

It is possible that classroom based mathematics builds on skills that are responsible for the association between mathematics with estimation ability; some of these early abilities may be more relevant for dot estimation skills (hence the association of dot estimation with teacher mathematics at 7 but not with GCSE). It is unclear whether the contemporaneous association between dot estimation and mathematics (both measured at age 16) was restricted to the Web assessed mathematics because of shared methods (e.g., both collected with online tests). Because Web assessed mathematics was not available at age 7, we cannot differentiate whether (1) web assessment taps into some abilities that emerge at a later age (age 16) and are important for estimation or (2) web assessments draw on some abilities unimportant for estimation. However, if shared methods were a source of association we should also observe the link of number line only with Web assessed mathematics and not with GCSE.

### Associations Among Estimation, Mathematics, and Related Cognitive Abilities Over Time

The third aim of the study was to examine whether the links between mathematical ability and estimation are present after accounting for a number of verbal and nonverbal abilities measured in the same children at 7, 9, 10, 12, 14, and 16 years of age.

First, mathematics at each age (Web, teacher assessed, GCSE scores at 16) were separately entered into multiple regressions as criterion variables. Number line and dot estimation were entered as predictors together with other cognitive abilities. The twins’ sex was also entered in each regression as predictor of mathematics and estimation (see Table 5).[Table-anchor tbl5]

In the presence of other cognitive abilities, number line estimation was a significant predictor of mathematics (teacher and Web assessed) at each age (average 11% of the variance explained). Conversely, dot estimation was a significant predictor of Web assessed mathematics at age 16 only, and of teacher assessed mathematics at ages 7, 9, and10 (average 5% of the variance explained). Other cognitive abilities explained between 7% (non-verbal abilities scores at age 7) and 32% (non-verbal ability scores at 16) of the variance in mathematics.

The next set of analyses examined whether estimation at 16 was best predicted by mathematics as opposed to other cognitive skills. Number line and dot estimation were entered as dependent variables, with mathematics and general cognitive abilities at ages 7, 9, 10, 12, 14, and 16 entered as independent predictors (see Table 6). Separate regressions were run for teacher rated (and GCSE) and Web assessed mathematics. The significance level for these regressions was adjusted for multiple testing (.05 ÷ 9 = .006, *p* < .01).[Table-anchor tbl6]

In the presence of other cognitive abilities, all measures of mathematics explained between 8% (age 7) and 17% (age 16) of the variance in number line estimation. However, other abilities were also significant predictors of number line estimation: non-verbal abilities at age 9, 10, and 16 (average 7% variance explained); reading at age 7 and 12 (average 6% of the variance; only when mathematics Web was included at age 12); spatial ability at age 12; and memory scores at age 16 (respectively explaining 8% and 5% of variance).

The pattern was overall similar for dot estimation, although its association with mathematics was uneven. Mathematics measured at age 7, 9, 10, and 12 was a significant predictor of dot estimation at age 16 (average variance explained 5%). At age 16 only Web assessed mathematics added independent variance (10%). After correction for multiple testing, other cognitive abilities explained independent variance in dot estimation: non-verbal abilities at age 9, 10, 14, and 16 (average 5%); reading at age 7 (2%); spatial ability at age 12 (4%, only when teacher assessed mathematics was included), and speed of processing scores at age 16 (4%. average contribution when GCSE and Web scores were included).

### Sex Differences in Estimation and Mathematics

As shown in Table 3, boys and girls performed very similarly on both measures of estimation assessed at age 16. Mean differences were significant for number line estimation only. However, the effects of sex on both estimation measures were negligible (η_p_^2^ = .00 for both). No meaningful variance or mean sex differences were observed for other measures. Sex was included as a predictor of mathematics at each age in all regressions presented in Table 5 and explained between 0% and 3% of the variance. When sex was included as a predictor of number line estimation and dot estimation, it was not a significant predictor (see Table 6). Further ANOVAs were conducted to assess the effects of sex on the two mathematics measures at age 16 after controlling for number line and dot estimation scores, separately for each measure. In these analyses, the partial eta-squared were almost identical (η_p_^2^ = .00 and .03 for GCSEs and Web scores respectively) to the partial eta-squared for the mathematics measures shown in Table 3. This suggests that the small sex differences observed in mathematics at age 16 may not be related to estimation.

## Discussion

This longitudinal study used a large U.K. representative sample of students to investigate number sense abilities and their association with mathematics. Specifically, the study examined the extent to which symbolic and nonsymbolic estimation abilities are associated to each other at the age of 16. It also investigated the relationship between these two aspects of estimation with concurrent and earlier mathematics achievement. The specificity and continuity of this relationship was assessed controlling for a number of cognitive abilities measured across the school years. A particular strength of the design was the employment of a discovery-replication approach, by generating two matching samples using one, randomly selected twin from each pair in each set of analyses.

A modest correlation (*r* = .22) was observed between symbolic number line and nonsymbolic dot estimation. This is similar to that of another study that used a sample of ∼11-year-old children (Fazio et al., 2014). A similar modest correlation (*r* = .28) was found in 5-year-olds (but not when the children were 4 and 6 years; Kolkman, Kroesbergen, & Leseman, 2013). This study used a nonsymbolic dot task (similar to the one used in Fazio et al.) and a number line task 0–100. Different dot tasks may tap into different aspects of nonsymbolic estimation (Mazzocco et al., 2011a). For example, completion of tasks that control for different visual cues in the dot display (e.g., cumulative surface area and dot size) may be driven by inhibitory control rather than numerical cues (e.g., Fuhs & McNeil, 2013; Gilmore et al., 2013). Furthermore, different dot task protocols may lead to different performance (Clayton et al., 2015). Indeed, the dot task protocols and stimuli were different in Fazio et al. (2014) and in our study. In the former, the two arrays of size and area controlled dots were presented separately, while in our dot task the display contained intermixed yellow and blue size-controlled dots. With our display the two numerosities always occupied the same area and it may be argued that response may have been driven by the visual property of the array (area, size of dots, or color) rather than numerical information. Despite such differences in protocol and stimuli our results are very similar to that of other reports. One possible reason is that if required, adults can suppress response on the basis of continuous properties of a stimulus (area) and respond to numerosity (Nys & Content, 2012).

In our study we further explored the relationship between the two estimation abilities, conducting an exploratory Factor Analysis on all measures collected at age 16. The results showed that number line and dot estimation loaded on a common (non-verbal ability) factor when the model allowed for only two factors. However, when the model became more flexible (3 factors), dot estimation loaded onto a separate factor, together only with speed of processing. The clustering of the dot task into a “speed factor” could reflect a measurement bias; for example, speed was required in completion of dot tasks and speed of processing trials but not number line trials. However, the mathematics test of problem verification was also timed but did not load on the third factor. This relative autonomy may stem from the fact that the number line test requires knowledge of formal symbolic representation of relative numerosity, whereas the dot task does not require such knowledge.

The degree of dissociation between number line and dot estimation could also be observed in the different patterns of association with mathematical ability. Retrospectively and prospectively, number line estimation at age 16 was significantly related to mathematics (both Web and teacher assessed) at each age, beyond variance explained by other cognitive abilities at that age. In these regressions the sample size ranged between 245 and 2219 at different ages. The association between number line estimation measured at 16 was detected in all samples. Conversely the association between mathematics and dot estimation was less consistent over time, supporting previous research (Chen & Li, 2014; Schneider et al., 2017).

Because dot estimation was only measured at age 16, it remains unclear whether the links between dot estimation and mathematics are stronger earlier in development. The observed developmental pattern is likely to reflect the heterogeneity of the mathematical domain. It is possible that when mathematics becomes more complex and abstract it may rely more strongly on spatial and other non-verbal cognitive abilities than on nonsymbolic estimation.

Other studies have suggested that different aspects of mathematics may be more closely related to dot estimation than others (e.g., Mazzocco et al., 2011a). Accordingly, our Web assessed mathematics at 16, but not school GCSE scores, correlated with dot estimation. Web assessed mathematics, which includes the component of fluency, correlated also with speed of processing at the same age, to which dot estimation was also correlated. This pattern of association between dot estimation, mathematical fluency, and speed of processing is of particular interest. Another study found that growth in nonsymbolic estimation abilities predicted mathematical fluency but not mapping or mathematical reasoning in first grade children (Toll, Van Viersen, Kroesbergen, & Van Luit, 2015). Previous research also suggests that automaticity in retrieval of the basic arithmetic facts (speed of processing) is important in mathematical learning (Bull & Johnston, 1997; Hitch & McAuley, 1991). It is possible that nonsymbolic estimation skills may be involved only at early stages of mathematics learning, in preschool or early school years, (e.g., Bonny & Lourenco, 2013; Mazzocco et al., 2011a), contributing to acquiring automaticity in basic arithmetic.

However, it remains unclear whether successful automaticity reflects foundational abilities that promote mathematics learning, or whether achieving automaticity supports later learning and therefore mediates the relationship between early estimation abilities and later mathematics. For example, once automaticity has been achieved, nonsymbolic estimation may no longer be necessary, and plays a less significant role in subsequent achievement gains. This idea is supported by one study that found no association between nonsymbolic estimation performance and mathematical ability in adults. Interestingly, however, individuals with higher mathematical skills had a more automatic access to nonsymbolic numerosity, reflected in slower performance on a numerical Stroop-like task (Nys & Content, 2012). In other words, individuals with higher mathematics performance were more impaired by an incongruent condition (mismatch between numerical and numerosity information), compared with people with lower maths performance. Presumably this was because nonsymbolic information was automatically activated in high maths performers, despite the irrelevance of this information for the task. The authors proposed that proficiency in nonsymbolic estimation skills may be connected with higher level of automaticity that is observed in people with higher mathematical skills. Our study may provide an indirect evidence for this: Beyond the contribution of concurrent mathematics, the earliest mathematics at age 7 added independent variance to dot estimation. This was our earliest measure of mathematics, when children begin to master mathematical symbols and rules and to build automated mathematical processing.

In our study, the association of symbolic and nonsymbolic estimation was not unique to mathematics; both estimation measures were associated with cognitive abilities beyond their associations with mathematics. For example, number line estimation at 16 was predicted by non-verbal ability at ages 9, 10, and 16, beyond mathematics at these ages. This is consistent with previous studies that reported associations of number line estimation with IQ (e.g., Bachot et al., 2005; Geary et al., 2008). Dot estimation at 16 was also predicted by non-verbal ability at ages 9 and 10, reading at age 7, and spatial ability at age 12. These results counter the view of nonsymbolic estimation as a numerical specific process. The results are consistent with recent reports of reduced but greater than zero correlations between nonsymbolic estimation and mathematics after controlling for inhibitory control in young children (that was not controlled for in our design; Keller & Libertus, 2015) and after controlling for other cognitive abilities (Chen & Li, 2014). Together with the results of the factor analysis, this evidence points to estimation as related to the multifaceted domains of intelligence (e.g., the three-stratum model, Carroll, 1993). Further research is needed to explore the nature of the specific associations observed in this study. For example, dot estimation at age 16 was uniquely (beyond mathematics and other abilities) associated with early reading (age 7) that was assessed by word recognition/decoding tests. Evaluation of small numerosity arrays containing 2 or 3 items (subitizing) is a perceptual process that may not require counting; Gelman and Gallistel (1978) note that in children, estimation of numerosities up to 6 can rely on recognition of patterns of the possible configuration of 2, 3, 4, 5, and 6 elements. As word recognition relies on pattern recognition, it is possible that the observed association between dot estimation and early reading is partially related to pattern recognition processes. Although the processes underlying nonsymbolic estimation need to be fully understood, studies have shown that estimation does not involve numerical processing exclusively but relies on other visual cues present in the stimuli (e.g., Clayton et al., 2015; Gebuis & Reynvoet, 2012). Pattern processing may be at the core of the correspondence between numerosity of a set and its symbolic representation of number, a process that has been proposed as vital for mathematical learning (Butterworth, 2005; Gelman & Gallistel, 1978). It is also possible that pattern recognition important in nonsymbolic estimation contributes to early number symbols learning, which in turn influences future mathematics achievement (van Marle et al., 2014).

Finally, the study tested whether the small but significant male advantage in mathematics found at age 16 was related to estimation. No meaningful sex differences were found in either of the two estimation tasks at age 16, suggesting that some early differences may disappear by this age. Therefore, the observed sex differences in mathematics at age 16 are not explained by estimation differences, but cognitive (e.g., spatial, Wei, Chen, & Zhou, 2016) or other noncognitive (e.g., academic anxiety; Wang et al., 2014). Further research is needed to understand whether the inconsistencies in the literature regarding sex differences in estimation are related to differences in samples, specific measures used, or developmental patterns.

The present investigation addressed “which, when, and how” questions about the relationship between number sense and mathematics. Although our study looked at the development of mathematics between the age of 7 and 16 years, estimation was measured only at age 16. This type of data has allowed only correlational analyses, limiting the understanding of directionality of effects. Also, the variability of the sample size and composition, together with the diverse measures of cognitive abilities and mathematics used, may have contributed to some of the uneven association of the dot task and mathematics. The results suggest that different measures of number sense at age 16 are partially independent constructs and are differentially related to mathematics. This supports the theory that symbolic and nonsymbolic estimation follow partially different developmental paths (Lyons, Ansari, & Beilock, 2012). More longitudinal research is needed to explore the directionality of the associations between different aspects of number sense and mathematical ability. As with other constructs related to mathematics, it is likely that the influences between estimation and mathematics are reciprocal (Carey, Hill, Devine, & Szücs, 2016).

Most covariance between mathematics and estimation was shared with other cognitive skills. Such results are consistent with previous research whereby a symbolic and a nonsymbolic task, although distinct from each other, contributed uniquely to mathematics achievement and the strength of their association with mathematics depended on the type of mathematical task (Mazzocco et al., 2011a). Importantly, much of the variance in participants’ estimation performance at age 16 remained unexplained, suggesting that estimation is a complex construct. At age 16, when estimation skills are relatively mature (Halberda et al., 2012), only 18% and 10% of the variance in number line and dot estimation respectively was explained by all other variables examined at this age. More research is needed to identify sources of the wide variability in estimation (Halberda et al., 2012).

Taken together, the results from this study indicate that relationship between number sense and mathematics depends on which specific aspects are considered and at which age.

## Supplementary Material

10.1037/dev0000331.supp

## Figures and Tables

**Table 1 tbl1:** Summary Measures from Age 7 to 16 Years

Domains assessed	Age 7	Age 9	Age 10	Age 12	Age 14	Age 16
Number Line estimation						Web
Dot estimation						Web
Mathematics	Teach. Quest.	Teach. Quest.	Teach. Quest.; Web	Teach. Quest.; Web	Teach. Quest.	Exams; Web
Verbal ability	Telephone	Child Quest.	Web	Web	Web	Web
Non-verbal ability	Telephone	Child Quest.	Web	Web	Web	Web
Reading fluency	Telephone			Web		Web
Reading comprehension			Web	Web		Web
Language				Web		Web
Spatial ability				Web		
Memory						Web
Speed of processing						Web
*Note*. Data gathered using the following: Telephone testing (Telephone); Child Questionnaire (Child Quest.); Teacher Questionnaire (Teach. Quest.); Web testing (Web); Exams results (Exams).						

**Table 2 tbl2:** Test Re-Test and Internal Validity Measures Age 16

	Test re-test on validation study		Cronbach alpha on all web data	
Measure	*r*	*N*	α	*n*
Number Line test	.70	45	.63	2534
Dot Task test (on accuracy)	.62	48	.74	2495
Mathematics fluency-Problem Verification Task test	.78	48	.85	2447
Mathematics-Understanding Numbers test	.67	48	.90	2352
Verbal ability (Vocabulary test)	.65	48	.82	2722
Non-verbal ability (Raven test)	.72	48	.80	2459
Reading fluency test	.81	48	.96	2548
Reading comprehension test	.67	48	.70	2114
Language test	.74	48	.69	2587
Speed of processing (accuracy on Reaction Time test)	.68	48	—	—
Speed of processing (time of response on Reaction Time task test)	.58	48	.95	2446
Memory (Corsi Tapping Block test)	.64	44	.69	2449
*Note*. *r* = test retest correlation; α = Cronbach alpha; *n* = sample size. In the validation study, 24 twin-pairs repeated all the web tests two months after the first web-assessment. Cronbach alpha is calculated on the data from all cohorts assessed on the web when the twins were 16 years old.				

**Table 3 tbl3:** Means, Standard Deviations, and Effects of Sex on Variables at Age 16

	Means and Standard deviations on raw data		Means and Standard deviations on standardised data						ANOVA-effects of sex		
	All		All		Females		Males		Sex		
Measure (scores on the test)	*M*	*SD*	*M* (*N*)	*SD*	*M* (*N*)	*SD*	*M* (*N*)	*SD*	*p*	η_p_^2^	*R*^2^
Number line estimation	36.66	15.62	−.01 (*n* = 2792)	.98	.03 (*n* = 1614)	.98	−.05 (*n* = 1178)	.98	.03	.00	.00
Dot estimation (Weber Fraction)	.28	.13	−.12 (*n* = 2437)	.76	−.11 (*n* = 1421)	.75	−.14 (*n* = 1016)	.76	.42	.00	.00
Mathematics GCSE scores	8.87	1.26	.03 (*n* = 5707)	.97	.01 (*n* = 3032)	.98	.06 (*n* = 2675)	.96	.01	.00	.00
Mathematics web scores	—	—	.02 (*n* = 2521)	1.0	−.13 (*n* = 1471)	.98	.23 (*n* = 1050)	.99	.02	.03	.03
Verbal ability scores	15.35	4.29	−.02 (*n* = 2697)	.95	−.02 (*n* = 1564)	.95	−.03 (*n* = 1133)	.95	.71	.00	.00
Non-verbal ability scores	13.86	3.77	.00 (*n* = 2449)	.97	−.02 (*n* = 1439)	.94	.03 (*n* = 1010)	1.0	.19	.00	.00
Reading composite scores	—	—	.01 (*n* = 2661)	.99	.02 (*n* = 1550)	.98	−.01 (*n* = 1111)	1.0	.53	.00	.00
Language scores	10.28	2.57	.02 (*n* = 2563)	.95	.03 (*n* = 1490)	.94	.02 (*n* = 1073)	.95	.74	.00	.00
Speed of Processing scores	37.56	1.82	−.06 (*n* = 2412)	.84	−.07 (*n* = 1404)	.82	−.04 (*n* = 1008)	.87	.00	.01	.00
Memory (Corsi) scores	5.50	2.03	.03 (*n* = 2445)	.97	−.03 (*n* = 1427)	.92	.13 (*n* = 1018)	1.0	.00	.01	.01
*Note*. *n* = sample size based on one randomly selected twin in the pair; *M* = mean; *SD* = Standard deviation; *p* = *p*-value of the effects of sex on variables; η_p_^2^= partial eta-squared; *R*^2^ = variance explained by sex. Standardized variables have been cleared of outliers scores (±3 standard deviations). Mean and standard deviation on raw data for the Number Line test represent the average error in estimation. The mathematics web test and reading scores are composites obtained by averaging the standardized means of two tests scores, therefore no raw data is provided for these composites. Descriptive statistics on speed of processing are presented for efficiency scores derived from the reaction time test; the column with raw data reports mean and standard deviation for accuracy on the test. Boys and girls showed different variance in non-verbal ability and memory (significant Levene’s test), however, these differences contributed to 1% of variance (*R*^2^) in memory and less than 1% in non-verbal ability.											

**Table 4 tbl4:** Regressions Method Forced Entry. Mathematics at Each Age Predicted by Number Line Estimation and Dot Estimation Measured at Age 16

	DV = Mathematics school achievement teacher rated & GCSE scores (age 16)		DV = Mathematics web test	
Model	β	*t*	β	*t*
Mathematics age 7 predicted by the scores				
Number line estimation	−.22	−9.36**		
Dot estimation	−.17	−7.09**		
	*R*^2^ = .10; *F*(2, 1651) = 86.64; *p* < .001			
Mathematics age 9 predicted by				
Number line estimation	−.26	−9.91**		
Dot estimation	−.15	−5.69**		
	*R*^2^ = .11; *F*(2, 1381) = 80.50; *p* < .001			
Mathematics age 10 predicted by				
Number line estimation	−.29	−11.73**	−.25	−10.58**
Dot estimation	−.11	−4.48**	−.15	−6.08**
	*R*^2^ = .11; *F*(2, 1477) = 92.38; *p* < .001		*R*^2^ = .10; *F*(2, 1653) = 91.11; *p* < .001	
Mathematics age 12 predicted by				
Number line estimation	−.27	−8.30**	−.31	−13.74**
Dot estimation	−.14	−4.14**	−.20	−8.82**
	*R*^2^ = .11; *F*(2, 894) = 54.38; *p* < .001		*R*^2^ = .16; *F*(2, 1768) = 166.72; *p* < .001	
Mathematics age 14 predicted by				
Number line estimation	−.34	−5.78**		
Dot estimation	−.11	−1.86		
	*R*^2^ = .14; *F*(2, 275) = 24.36; *p* < .001			
Mathematics age 16 predicted by				
Number line estimation	−.31	−15.11**	−.35	−18.28**
Dot estimation	−.16	−7.90**	−.23	−11.81**
	*R*^2^ = .15; *F*(2, 2104) = 179.60; *p* < .001		*R*^2^ = .21; *F*(2,2259) = 298.50; *p* < .001	
*Note*. DV = Dependent variable; β = standardized beta; *t* = *t*-value of β, significant t-values are reported in bold characters. Regressions based on one randomly selected twin in each pair. Analyses were repeated using the second half of the sample, with very similar results (available from the authors).				
** *p* < .001.				

**Table 5 tbl5:** Summary of Multiple Regressions, Method Forced Entry. Mathematics Predicted at Each Age by Number Line and Dot Estimation Measured at Age 16 and Cognitive Abilities Measured at the Same Age of Mathematics

	DV = Mathematics school achievement: teacher rated & GCSE scores (age 16)			DV = Mathematics web tests		
Model	β	*t*	η_p_^2^	β	*t*	η_p_^2^
Mathematics age 7 predicted by						
Number line estimation at 16	−.12	−5.51***	.08			
Dot estimation at 16	−.09	−4.41***	.05			
Verbal ability at 7	.15	6.37***	.15			
Non-verbal ability at 7	.08	3.70***	.07			
Reading at 7	.41	17.75***	.29			
Sex	.07	3.25***	.00			
	*R*^2^ = .34; *F*(6, 1526) = 130.84; *p* < .001					
Mathematics age 9 predicted by						
Number line estimation at 16	−.17	−6.40***	.09			
Dot estimation at 16	−.11	−4.41**	.04			
Verbal ability at 9	.17	6.35***	.10			
Non-verbal ability at 9	.23	8.31***	.15			
Sex	.07	2.92**	.00			
	*R*^2^ = .20; *F*(5, 1257) = 43.00; *p* < .001					
Mathematics age 10 predicted by						
Number line estimation at 16	−.20	−7.33***	.09	−.12	−5.90***	.09
Dot estimation at 16	−.07	−2.75**	.03	−.04	−1.93	.04
Verbal ability at 10	.15	4.75***	.13	.20	8.09***	.28
Non-verbal ability at 10	.11	3.60**	.11	.31	13.16***	.31
Reading at 10	.19	6.02***	.13	.20	8.35***	.26
Sex	.05	◆ 1.76	.00	.04	1.96*	.01
	*R*^2^ = .23; *F*(6, 1166) = 58.62; *p* < .001			*R*^2^ = .41; *F*(6, 1520) = 177.66; *p* < .001		
Mathematics age 12 predicted by						
Number line estimation at 16	−.09	−2.46**	.11	−.11	−5.30***	.13
Dot estimation at 16	−.06	−1.67	.04	−.06	◆ 2.98**	.07
Verbal ability at 12	.09	2.00*	.17	.11	4.27***	.28
Non-verbal ability at 12	.05	◆1.25	.12	.20	8.61***	.28
Reading at 12	.25	6.20***	.22	.24	9.98***	.31
Language at 12	.17	3.74***	.19	.20	7.99***	.31
Spatial ability at 12	.09	◆2.46**	.08	.14	6.71***	.21
Sex	−.01	−.21	.00	.05	◆2.71**	.00
	*R*^2^ = .30; *F*(8, 647) = 35.97; *p* < .001			*R*^2^ = .52; *F*(8, 1401) = 190.26; *p* < .001		
Mathematics age 14 predicted by						
Number line estimation at 16	−.23	−4.17***	.15			
Dot estimation at 16	.00	.01	.05			
Verbal ability at 14	.29	5.16***	.23			
Non-verbal ability at 14	.32	5.58***	.23			
Sex	.11	2.21*	.00			
	*R*^2^ = .39; *F*(5, 237) = 32.07; *p* < .001					
Mathematics age 16 predicted by						
Number line estimation at 16	−.14	−7.27***	.12	−.19	−11.20***	.17
Dot estimation at 16	−.02	−.97	.06	−.08	−4.11***	.10
Speed of processing at 16	−.09	−4.52***	.10	.10	−5.92***	.11
Memory (Corsi)) at 16	.11	5.83***	.11	.10	5.77***	.14
Verbal ability at 16	.13	6.09***	.19	.08	4.36***	.17
Non-verbal ability at 16	.21	10.39***	.24	.27	14.98***	.32
Reading at 16	.19	8.62***	.25	.13	6.33***	.23
Language at 16	.16	7.47***	.24	.18	9.14***	.27
Sex	.02	◆ 1.27	.00	.17	10.79***	.03
	*R*^2^ = .43; *F*(9, 1830) = 155.52; *p* < .001			*R*^2^ = .51; *F*(9, 2038) = 232.66; *p* < .001		
*Note*. DV = Dependent variable; β = standardized beta; *t* = *t*-value of β, significant t-values are reported in bold characters; η_p_^2^ = partial eta-squared. The symbol ◆ indicates results significant in one sample of twins and non-significant in the co-twins. Regressions based on one randomly selected twin in each pair.						
* *p* < .05. ** *p* < .01. *** *p* < .001.						

**Table 6 tbl6:** Summary of Multiple Regressions, Method Forced Entry. Number Line, and Dot Estimation Measured at Age 16 Predicted by Mathematics and Cognitive Abilities Measured at Age 16 and at Previous Ages

	DV = Number line estimation at age 16			DV = Dot estimation at age 16		
Model	β	*t*	η_p_^2^	β	*t*	η_p_^2^
Age 7 predictors of estimation						
Mathematics teacher at 7	−.21	−7.43***	.08	−.18	−5.95***	.05
Verbal ability at 7	−.03	−1.06	.02	.01	.25	.01
Non-verbal ability at 7	−.04	−1.80	.02	−.04	−1.62	.01
Reading at 7	−.08	−3.05**	.05	−.08	−2.73**	.02
	*R*^2^ = .08; *F*(5, 1746) = 32.17; *p* < .001			*R*^2^ = .06; *F*(5, 1546) = 19.40; *p* < .001		
Age 9 predictors of estimation						
Mathematics teacher at 9	−.22	−8.07***	.09	−.17	−5.74***	.04
Verbal ability at 9	−.01	−.50	.02	.05	1.83	.00
Non-verbal Ability at 9	−.13	−4.45***	.05	−.15	−5.06***	.03
	*R*^2^ = .09; *F*(4, 1423) = 37.46; *p* < .001			*R*^2^ = .06; *F*(4, 1270) = 21.37; *p* < .001		
Age 10 predictors of estimation						
Mathematics teacher at 10	−.22	−7.79***	.09	−.13	−4.00***	.03
Verbal ability at 10	−.03	−.96	.05	.03	.99	.02
Non-verbal ability at 10	−.11	−3.52***	.06	−.14	−4.23***	.04
Reading at 10	−.06	−1.76	.05	−.05	−1.34	.03
	*R*^2^ = .11; *F*(5, 1311) = 33.30; *p* < .001			*R*^2^ = .05; *F*(5, 1178) = 13.09; *p* < .001		
Age 10 predictors of estimation						
Mathematics web at 10	−.21	−7.06***	.09	−.10	−3.01***	.04
Verbal ability at 10	−.03	−1.03	.05	−.01	−.38	.02
Non-verbal ability at 10	−.08	−2.82**	.06	−.13	−3.98***	.04
Reading at 10	−.06	◆ −2.04*	.05	−.05	−1.46	.03
	*R*^2^ = .10; *F*(5, 1717) = 40.17 *p* < .001			*R*^2^ = .05; *F*(5, 1537) = 17.17; *p* < .001		
Age 12 predictors of estimation						
Mathematics teacher at 12	−.14	−3.45**	.11	−.09	−2.03*	.04
Verbal ability at 12	−.05	−1.14	.06	−.02	−.42	.03
Non-verbal ability at 12	−.13	◆ −3.13**	.07	−.05	−1.09	.04
Reading at 12	−.09	◆ −2.15*	.06	−.02	−.52	.04
Language at 12	−.04	−.83	.05	−.05	−.90	.03
Spatial ability 12	−.10	−2.64**	.08	−.12	−2.85***	.04
	*R*^2^ = .16; *F*(7, 723) = 20.89; *p* < .001			*R*^2^ = .05; *F*(7, 655) = 6.80; *p* < .001		
Age 12 predictors of estimation						
Mathematics web at 12	−.20	−6.13***	.13	−.15	−4.05***	.07
Verbal ability 12	−.05	−1.50	.06	−.04	−1.01	.03
Non-verbal ability at 12	−.06	−1.94	.07	−.08	◆ −2.50*	.04
Reading at 12	−.10	−3.22**	.06	−.06	◆ −1.86	.04
Language at 12	.05	1.48	.05	.03	.77	.03
Spatial ability at 12	−.11	−3.84**	.08	−.05	−1.54	.04
	*R*^2^ = .14; *F*(7, 1587) = 36.47; *p* < .001			*R*^2^ = .07; *F*(7, 1414) = 16.51 *p* < .001		
Age 14 predictors of estimation						
Mathematics teacher at 14	−.31	−4.47***	.15	−.08	−1.12	.05
Verbal ability at14	−.11	−1.66	.06	−.09	−1.24	.04
Non-verbal 14	−.08	−1.24	.08	−.21	−2.99***	.06
	*R*^2^ = .17; *F*(4, 267) = 15.22; *p* < .001			*R*^2^ = .09; *F*(4, 240) = 6.89; *p* < .001		
Age 16 predictors of estimation						
Mathematics GCSE at 16	−.21	−7.85***	.12	−.05	−1.87	.06
Verbal ability at 16	.04	1.62	.03	−.02	−.80	.03
Non-verbal ability at 16	−.14	−5.52***	.09	−.13	−4.82***	.07
Reading at 16	−.04	◆ −1.52	.06	.00	◆ .05	.03
Language at 16	−.04	−1.68	.06	−.06	◆ −2.22*	.05
Speed of processing at 16	−.05	2.43	.04	.17	7.23***	.07
Memory (Corsi) at 16	−.08	−3.57***	.05	−.05	◆ −2.00**	.03
	*R*^2^ = .15; *F*(8, 1973) = 44.83; *p* < .001			*R*^2^ = .08; *F*(8, 1841) = 20.95; *p* < .001		
Age 16 predictors of estimation						
Mathematics web at 16	−.32	−12.17***	.17	−.15	−5.34***	.10
Verbal ability at16	.04	1.41	.03	−.02	−.65	.03
Non-verbal ability at 16	−.08	−3.41**	.09	−.09	−3.39**	.07
Reading at 16	−.05	◆ −1.83*	.06	.02	◆ −.87	.03
Language at 16	.00	−.16	.06	−.05	−1.84	.05
Speed of processing at 16	.04	−1.94	.04	.17	7.44**	.01
Memory (Corsi) at 16	−.07	−3.39**	.05	−.05	◆−2.29*	.03
	*R*^2^ = .18; *F*(8, 2210) = 61.08; *p* < .001			*R*^2^ = .10; *F*(8, 2057) = 29.33; *p* < .001		
*Note*. DV = Dependent variable; β = standardized beta; *t* = *t*-value of β, significant t-values are reported in bold characters; η_p_^2^ = partial eta-squared. The symbol ◆ indicates results significant in one sample of twins and non-significant in the co-twins. Regressions based on one randomly selected twin in each pair. Sex was included as a predictor in each model. However, as it did not significantly predict number line or dot estimation, the measure is not shown in this table. The number of degrees of freedom reflects the presence of sex as variable in the regression.						
* *p* < .05. ** *p* < .01. *** *p* < .001. *p*-values < .05 are not considered significant after correction for multiple testing.						
