# Supplementary material for: Number Sense and Mathematics: Which, When and How?
Source: Dev Psychol. 2017 Jul 31;53(10):1924–39. doi: 10.1037/dev0000331 (PMC5611774; doi:10.1037/dev0000331)
Supplement: Supplementary file 1 [file z2p999173929so1.docx]

**Supplemental Materials**

**Number Sense and Mathematics: Which, When and How?**

**by M. G. Tosto et al., 2017, *Developmental Psychology***

**http://dx.doi.org/10.1037/dev0000331**

**Method**

**Recruitment sample and testing procedure at age 16**

TEDS 16-year online assessment was carried on two of the three twins' cohorts. Families with twins born in 1994 and 1995 were sent a postal invitation, which included information about the web-based study and details to access TEDS testing website. Parents gave online consent for their family by using their secure ID and password to access the website. The twins’ log-ins were activated after the parents' consent. TEDS families are accustomed with this procedure because they have previously taken part in web-based studies when the twins were aged 10, 12 and 14 years of age (see for example Haworth et al., 2007). The web–tests were designed to be carried out without supervision; each test had a set of instructions, an online tutorial and practice trials where appropriate. In order to maximise the twins’ participation, it was possible to pause some of the tests and/or complete the battery in more than one session. If required, a dedicated team of testers telephoned the families to encourage the twins to complete the activities.

GCSE (General Certificate of Secondary Education) scores were collected from the three cohorts of twins soon after the exams results were available from the schools, between August 2010 and August 2013. The results forms were mailed to the families and were completed by the parents or the twins themselves.

**Homogeneity of the samples over time**

Although all participants were drawn from the TEDS study, data were not always collected from all twins at all time points. Therefore, samples at each age were only partially overlapping. For example, because of budget constraints at age 16 the tests online were administered only to cohorts 1 and 2, but questionnaires collecting various behavioral data and school achievement were sent to the 3 cohorts. At age 14, teacher's data (which provides measurement of school achievement comparable to previous assessments) were collected only from the 1^st^ cohort as the focus of the study was on parental measures. Further, missing data due to attrition or temporary inactivity lead to different sample compositions at each wave of testing. However, data based on response rate at each assessment, show that, throughout the 20 years of the TEDS study, the sample has maintained representativeness of the UK population (Haworth, Davis, & Plomin, 2013; Oliver & Plomin, 2007). This in turn suggests comparability of the samples at each data collection. However, in order to ensure that results of this study were not affected by attrition, we assessed homogeneity of the samples based on family socio-economic status (SES) computed from variables collected when the twins were about one and half years of age. The variable related to parent qualifications and employment and mother's age at birth of the first child. One way ANOVA using wave of testing as factor (age group at 6 levels) and SES at first contact as dependent variable reveled just a few significant but very small differences in mean and variance SES among the age groups (Table S1).These differences are more likely to be detected in virtue of the large sample size (n ranged between 3206 and 6740).

| Table S1 | | | | | | | | | | | |
| --- | --- | --- | --- | --- | --- | --- | --- | --- | --- | --- | --- |
| *Mean SES and sample size at each wave of testing. Comparison of significant mean SES differences* | | | | | | | | | | | |
|  | n | M | SD | Levene' test | | t-test | | | r effect size of difference | Cohen's d effect size of difference. | ƞ^2^_p_ effect size for significant Levene's test |
|  |  |  |  | F | p-value | t-value | df | p-value |  |  |  |
| **Group age 7** | **6740** | **.08** | **.71** |  |  |  |  |  |  |  |  |
| *Age 7 and group age 12* |  |  |  | 766.67 | .00 | -5.43 | 10778.41 | .00 | .05 | -.10 | .057 |
| *Age 7 and group age 14* |  |  |  | 670.54 | .00 | -8.27 | 4802.92 | .00 | .12 | -.24 | .063 |
| *Age 7 and SES age 16* |  |  |  | 712.00 | .00 | -7.49 | 10030.82 | .00 | .07 | -.15 | .055 |
|  |  |  |  |  |  |  |  |  |  |  |  |
| **Group age 9** | **3457** | **.11** | **.99** |  |  |  |  |  |  |  |  |
| *Age 9 and group age 14* |  |  |  | 1.33 | .25 | -5.84 | 6661 | .00 | .07 | -.14 | n/a |
| *Age 9 and group age 16* |  |  |  | .52 | .47 | -4.17 | 9069 | .00 | .04 | -.09 | n/a |
|  |  |  |  |  |  |  |  |  |  |  |  |
| **Group age 10** | **3494** | **.12** | **.98** |  |  |  |  |  |  |  |  |
| *Age 10 and group age 14* |  |  |  | 3.24 | .07 | -4.84 | 6698 | .00 | .06 | .12 | n/a |
| *Age 10 and group age 16* |  |  |  | .00 | .99 | -3.41 | 9106 | .00 | .04 | -.07 | n/a |
|  |  |  |  |  |  |  |  |  |  |  |  |
| **Group age 12** | **5986** | **.16** | **.93** |  |  |  |  |  |  |  |  |
| *Age 12 and group age 16* |  |  |  | 2.38 | .12 | -3.66 | 9190 | .00 | .04 | -.08 | n/a |
|  |  |  |  |  |  |  |  |  |  |  |  |
| **Group age 14** | **3206** | **.24** | **.99** | -- | -- | -- | -- | -- | -- | -- | n/a |
| **Group age 16** | **5614** | **.20** | **.99** | -- | -- | -- | -- | -- | -- | -- | n/a |
| Note: SES = socio economic status measured when the twins were ~1.5 years-old.; n = sample size of participants with available SES at that age. M = Mean for SES; SD = Standard deviation; F= F-value for the Levene's test in the independent sample t-test; t-value = t values from independent sample t-test; df= degrees of freedom; r = effect size of the significant SES mean differences. Cohen's d = magnitude of the effect size in SES mean differences. ƞ^2^_p_ = partial eta-squared of the effect size due to inequality of variance detected by the Levene's test. n/a = not applicable because the non-significant Levene's test suggests equality of variance. The table shows means and standard deviation for SES at each age in bold characters. The comparison between significantly different SES is reported in normal characters. There are no differences between mean SES in the group at age 14 and age 16. | | | | | | | | | | | |

In Table S1 we provide the magnitude of the effects size in the mean SES differences using the Pearson correlation '*r* ', which ranged between .03 and .12, and using Cohen's '*d*' which ranged between .07 and .24. *r* = .10 are considered small, as they explain 1% of the total variance. Cohen's *d* up to .20 are also considered small effects (Cohen, 1992). The significant mean SES differences between the groups at age 7 with the groups at age 12, 14 and 16, showed also inequality of variance (significant Levene's test, Table S1). However, variance differences explained 6% of the total variation (indexed by the partial eta-squared (ƞ^2^_p_) of the effect size due to inequality of variance detected by the Leven's test (Table S1).

**Testing differences in beta coefficients**

The regressions summarised in Table 4 (main manuscript) show number line and dot estimation used together in nine regressions as predictors of current (age 16) and earlier mathematics. The variability of these associations has been interpreted by evaluating the overlap of the 95% confidence intervals (CI) of the beta coefficient of number line and dot estimation derived from these nine regressions. Standardised beta coefficients are considered significantly different (p < .05) if their intervals overlap less than 50% the length of one CI arm (Cumming, 2009). Figure S1 depicts the standardised beta coefficients of the regressions summarised in Table 4 for number line estimation as predictor.

*Figure S1: Standardised beta coefficient for number line used as predictor of the 9 mathematics variables together with dot estimation (regressions in Table 4 main manuscript). M.T and M.W index mathematics teacher and web assessed respectively. For example, M.T_7 = teacher assesses mathematics at age 7, M.W_10 = web assessed mathematics at age 10.The bars represent the 95% confidence intervals (CI). Values of the beta coefficients, upper and lower estimates of the 95% CI are reported below each bar.*

It can be observed that the 95% CI of the beta coefficient of teacher assessed mathematics at age 7 greatly overlap with betas of teacher assessed mathematics at age 9, 12, 14 and mathematics web assessed at age 10. These beta coefficients can be considered statistically non-significantly different from each other. Conversely, one arm interval (from midpoint to the lower end) of mathematics at age 7 clearly overlaps less than 50% with one CI arm of from mathematics web assessed at 12, while there is no overlap with mathematics (web and GCSE) at age 16. These beta coefficients can be therefore considered statistically significantly different. The betas of teacher assessed mathematics at age 7 and 10, seem to overlap approximately 50%. In this case, half of the mean of the overlapping CI arms was computed as .024 (half of average of the difference between the low estimate (-.265) and the midpoint beta (-.224) of mathematics age 7, and the upper estimate (-.240) and beta (-.293) of mathematics teacher age 10; [((-.224)-(-.265)+(-.293)-(-.240))/2]/2 = 0.0235) and subtracted from the lower estimate of the beta coefficient of mathematics at age 7: [-.265- (.024)] =-.241. The upper estimate of math at age 10 is -.240, therefore the overlap of the two CI half arms is just over 50% indicating that the estimates are non-significantly different.

Applying the same reasoning, it can be observed that mathematics at age 9 is statistically significantly different only from mathematics web assessed at age 16.

Mathematics teacher assessed at age 10 is not significantly different from any other mathematics. Mathematics web at age 10 is significantly different only from mathematics at age 16 (both web and GCSE).

Beta for mathematics teacher assessed at age 12 is not significantly different from other betas except for mathematics web age 16, while beta for mathematics web assessed at age 12 is significantly different only from the beta of mathematics at age 7.

Beta for mathematics at age 14 is not significantly different from any other beta.

As mentioned in the previous results, beta for mathematics GCSE at 16 is significantly different only from mathematics at age 7, and mathematics web assessed at 10. Mathematics web 16 is significantly different from early teacher assessed mathematics at age 7, 9 and only web assessed at 10 and 12.The overlap between mathematics GCSE and mathematics teacher assessed at age 10 seems about 50%; however, subtracting half of the mean of the overlapping CI arms (0.021) from the lower estimate of the CI for mathematics at age 10 (-.164 - (.021)) = - .143, it shows that the overlap among the half-arms is over 50% as the upper estimate for the GCSE CI is -.130, thus suggesting that these two estimates are not significantly different from each other. Similarly, the betas of the two mathematics measures at age 16 are not significantly different from each other. Subtracting half of the mean of their overlapping CI-arms (0.021) from the lower estimate of the CI for mathematics GCSE (-.203 - (.021)) = -.182, it shows that their overlap is just over 50% as the upper estimate of the CI for web assessed mathematics at age 16 is -.181.

Overall, the beta coefficient for number line and mathematics at all ages are largely the same. When these are significantly different, the differences are more likely to occur when the mathematics measures are temporally more apart.

When the predictor of mathematics is dot estimation (in presence of number line estimation), the strength of the association of dot estimation, with mathematics is overall very similar over time. Standardised betas for mathematics up to age of 14 are not significantly different from each other (Figure S2). Teacher assessed mathematics at age 14 is significantly different from mathematics web assessed at age 16 but not from mathematics GCSE.

*Figure S2: Standardised beta coefficient for dot estimation used as predictor of the 9 mathematics variables together with number line estimation (regressions in Table 4 main manuscript. The bars represent the 95% confidence intervals (CI). Values of the beta coefficients, upper and lower estimates of the 95% CI are reported below each bar.*

Figure S3 depicts beta coefficients with their 95% CI for the regression where number line estimation is predicted by the contemporaneous mathematics (web assessed and GCSE at 16) and the earliest (teacher assessed at age 7). The betas of the two measure of mathematics at age 16 are not significantly different from each other, while they are both significantly different from the beta of mathematics at age 7.

*Figure S3 Standardised beta coefficient for number line predicted by the earliest mathematics (teacher assessed at age 7) and contemporaneus (web assessed and GCSE at age 16).The bars represent the 95% confidence intervals (CI). Values of the beta coefficients, upper and lower estimates of the 95% CI are reported below each bar.*

The same pattern is observed when dot estimation is predicted by contemporaneous mathematics (web assessed and GCSE at 16) and the earliest (teacher assessed at age 7). Figure S4 depicts betas with their 95% CI for this regression and it can be observed that betas for two measures of mathematics at age 16 are not significantly different from each other, while they are both significantly different from the beta of mathematics at age 7

*Figure S4: Standardised beta coefficient for dot estimation predicted by the earliest mathematics (teacher assessed at age 7) and contemporaneus (web assessed and GCSE at age 16).The bars represent the 95% confidence intervals (CI). Values of the beta coefficients, upper and lower estimates of the 95% CI are reported below each bar.*

**Quantile Regression analyses**

Quantile regressions were conducted to evaluate the strength of the association between estimation and mathematics across the low, medium and high distribution of abilities and whether similar patterns of association would be observed over time in these ability levels. Quantile regression assesses the relationship between a predictor and conditional quantiles of the outcome variable, rather than the conditional mean of the outcome. In other words, it assesses whether independent variables of interest have different effects along the distribution of the outcomes. In this study quantile regressions were conducted using the package 'quantreg' (Koenker, 2016) running in R environment (R Core Team, 2014). These analyses were conducted as follows:

(1) Two models were run, where mathematics at age 16 was used as outcome variables (web test assessed and GCSE separately), and the two estimation measures and cognitive abilities at age 16 as predictors. These two regressions assessed the strength of the association between the two mathematical measures at age 16 and the nine predictors at age 16 at: low - 25^th^, medium - 50^th^ and high - 75^th^ quantiles of the distribution of mathematics.

(2) Number line estimation and dot estimation were used separately as outcome variables with mathematics and cognitive abilities at age 7, 9, 10, 12, 14 and 16 as predictors. When in the same wave of assessment mathematics was measured with both web tests and teacher rating, two separate models were run using one the two mathematics measures at the time. For example, at age 10, number line was used as outcome variable, first in a model with predictors: verbal ability, non verbal ability, reading and mathematics assessed by teacher; and in a second model with mathematics web assessed. The same was done for dot estimation. These eighteen regressions assessed the strength of the association between the two estimation measures at the three quantiles (25^th^, 50^th^ and 75^th^) and mathematics and cognitive abilities, over 10 years’ time.

The twenty quantile regressions generated 124 graphs plotting changes in beta coefficients separately for each independent predictor. Here we present two samples of these plots. All the 124 graphs are available from the authors on request. Figure S5 is derived from the model where mathematics GCSE at age 16 is used as outcome and all the variables at age 16 as predictors. The plot summarises the effects of dot estimation along the distribution of the GCSEs scores; these are represented by the beta coefficient of the quantile regression (the dotted black line) with its 95% confidence intervals- CI (the grey area). The continuous horizontal red line and the red dotted lines represent respectively the mean beta value with 95% CI obtained with the least squares method.

*Figure S5: Plot of the changes in beta coefficients along the mathematics GCSE quantiles as function of dot estimation. Dot estimation is used as predictor of mathematics GCSE together with all 9 predictors at age 16. The x-axis indicates the quantiles, beta values are plotted on the y-axis. The dotted black line represents betas derived from the quantile models, the shaded grey area represents the 95% CI for quantile betas. The horizontal red line represents the beta values from the least squares model (β = -.02), the parallel dotted red lines are the 95% CI.*

The beta estimates, and related CIs, from both least squares and quantiles, are almost totally overlapping suggesting no significant differences in the relationship between dot estimation and mathematics GCSES for the whole distribution of abilities. Figure S6 presents the plot of the effects of number line estimation on mathematics GCSE derived from the quantile multiple regression model with all the variables at age 16 predictors of mathematics GCSE.

*Figure S6: Plot of the changes in beta coefficients for number line estimation as predictor of mathematics GCSE at age 16. Number line estimation is used as predictor of mathematics GCSE together with the 9 predictors at age 16. The x-axis represents the quantiles, beta values are plotted on the y-axis. The dotted black line represents betas derived from the quantile models, the shaded grey area represents the 95% CI for quantile betas. The horizontal red line represents the beta values from the least squares model (β = -.14), the parallel dotted red lines are the 95% CI.*

Significant differences in beta coefficient are present in the lowest quantiles, suggesting that below the ~10^th^ quantile the effects of the number line estimation on mathematics GCSE are significantly stronger than in the rest of the distribution. Similarly, significant differences were detected around the 80^th^ percentile, suggesting effects of number line estimation on GCSE significantly weaker than in the rest of the sample. Overall, for all the regressions, predictors had very similar effects on the outcomes along the whole of their distribution. Some differences were observed in some samples, whereby at the higher end of verbal and non-verbal, ability slightly stronger association with estimation were observed. As this pattern was consistent across the partially overlapping samples almost at all ages, it suggests that effects detected are not due to sample composition. A stronger association of early mathematics (ages 7-10) and dot estimation was also observed the high end of mathematical ability. These are effects that warrant further investigation.

As the effects of all predictors on the outcome variables of interest were largely the same along the distribution of abilities at all ages, for the purpose of this study, the results of the normal (least squared) regression can be used for the whole samples.

**Measures age 16**

The web based battery comprised of 11 tests detailed below.

**Number Line** - Task assessing estimation of numerical magnitudes, programmed and implemented on-line from a description in Opfer & Siegler ([2007](#_ENREF_16)). A line, with the left edge marked with “0” and the right edge marked with “1000” was presented in the middle of the screen with a numeral above it. The task required participants to indicate where they thought the target numeral should be, by dragging and releasing a red cursor along the line. Twenty-two numbers to be estimated were presented in the same order to all participants: 246, 179, 818, 78, 722, 150, 366, 122, 738, 5, 147, 938, 18, 606, 2, 34, 754, 100, 56, 163, 486, and 725. The choice of a fixed order was driven to minimise advantages, favoring accuracy that may have been produced by easier random presentations (where numbers in the same range were presented in close trials, for example). The trials were deemed age appropriate as at 16 years most children rely on linear representations of numerical magnitudes on a mental number line with numeral within a range 0-1000 (e.g. [Booth & Siegler, 2006](#_ENREF_1); [Siegler & Booth, 2004](#_ENREF_19)). Total length of the line was 500 pixels with each unit 0.5 pixels long, therefore accuracy in response recorded to the nearest 0.5 units. Participants' response was converted into a number estimated based on the number of pixels included between 0 and the mark made on the line. Scores were calculated as the mean error between targets and numbers estimated. This test allowed only one practice trial to reduce the effects of training/learning as this has been shown to positively affect estimation accuracy. Participants were given the option to continue with the task or to resume it later at any time in the test. The program recorded the scores as described as well as response reaction time.

**Dot Task** - The task assessing the ability to discriminate large numerosities was a shorter version (150 dot-size controlled trials) of the task described in Halberda, Mazzocco and Feigenson ([2008](#_ENREF_5)). The stimuli consisted of static pictures with mixed arrays of yellow and blue dots. The number of dots ranged between 5 and 21 for each colour with ratios organized in 8 bins with the lowest ratio of each bin serving as the top boundary of the following bin. The bins-ratio were organized as follow: 11 trials with a ratio randomly chosen between 8/7 and 7/6; 26 trials between 7/6 and 6/5; 28 trials between 6/5 and 5/4; 29 trials between 5/4 and 4/3; 26 trials between 4/3 and 3/2; 18 trials between 3/2 and 2; 8 trials between 2 and 3; 4 trials between 3 and 4. In all trials the average diameter of yellow dots was equal to the average diameter of blue dots. With this display, the set with more dots has a larger area on screen. Such design may have induced responses to the stimuli on the basis of visual properties of the stimuli, in addition to numerosity. However, studies have shown that, if required, adults can suppress response on the basis of continuous properties of a stimulus (area) and respond to numerosity ([Nys & Content, 2012](#_ENREF_15)). Prior to the online adaptation of this task, we conducted a pilot study (described in the section below) using the same task as in Halberda et al. (2008) which included 250 area and size controlled trials. Because of the length of the online battery, we needed to shorten the task, from 250 to 150 trials. The decision to control for size only in a version with 150 trials was taken on the basis of previous unpublished studies conducted by the authors of the Dot Task (Halberda et al., 2008). These studies showed no changes in individuals' Weber Fractions if this was calculated on trials controlled for area only, size only or both provided that the task was able to measure fine grain individual variation. Greater sensitivity of our task was achieved by increasing the number of ratios, from 3 (across 250 dot size and area controlled trials used in Halberta el al., 2008 task) to 8 ratios in 150 dot size-controlled trials.

The test was administered in a random sequence fixed for all participants to reduce eventual disadvantages and fatigue deriving from random sequences where more difficult trials were presented consecutively or towards the end of the test. Each stimulus flashed on the screen for 400ms, and participants had to decide whether the array contained more yellow or blue dots. Response was given by pressing “Y” for more yellow and “B” for more blue dots. Maximum allowed response time was 8 sec. If no answer was given during this time, the answer was recorded as wrong and a message appeared on the screen to encourage pressing the space bar to see the next trial. The message disappeared after 20 seconds and the next trial was displayed only after a press of the space-bar. There was a two items practice trial, with feedback and an option to repeat the practice if necessary. At the end of the practice trial it was made clear that the task measured speed as well as accuracy and invited participants to respond as quickly as possible. The task was divided in three blocks of 50 trials. At the end of each block it was possible to take a break and resume the test later. The test recorded accuracy and reaction time for each trial. A Weber Fraction score for each individual was derived using the method described in the supplementary information of Halberda et al. (2008). In addition, reaction time on response was used for a further correction. The Weber Fraction for each participant was derived only on trials not considered outliers according to the Jolicoeur method ([Van Selst & Jolicoeur, 1994](#_ENREF_23)). On average, 3.9 trials were removed from each performance, with a minimum of 0 and a maximum of 10. Weber Fraction is an amodal index of the ability to perceive changes in the appraised measure. In this study, the Weber Fraction indexed the ability to discriminate between the numbers of yellow and blue dots, with larger differences between the two arrays (i.e. larger ratios) being easier to discriminate. For example, given two arrays, one with 4 dots and one with 6, (ratio 2:3) the Weber Fraction is derived by dividing the difference of the two ratios by the smallest number in the ratio: [(3 - 2)/2] = 0.5.

**Problem Verification Task** - Assessed mathematical fluency and it was an online adaptation of a shortened version (from 88 to 48 items) of the task described in Murphy and Mazzocco ([2008](#_ENREF_11)). The selection of the items was based on correlation and reliability analysis conducted on data of a previous pilot study described in the section below. The reduced test of 48 items correlated .97 with the full-length version. The items were arithmetic problems (24 fraction problems and 6 problems each for: addition, multiplication, subtraction, and division), appearing on the screen one at a time with an answer provided. This is an example of a trial: 76 ÷ 7 = 10. The task was to judge as quickly as possible and within 10 seconds, whether the answer was correct or not. The proposed answer was correct in half of the trials. Response was given by pressing the keys F, J or K respectively for “correct”, “incorrect”, and “don’t know”. For every item, a reminder of which keys to press was shown at the bottom of the screen. A time bar on the top- left corner of the screen alerted participants of the elapsing time. If no answer was given during this time the next trial followed. The next item was presented immediately following a response. One point was awarded for each correct response. Timed out and “don’t know” responses received zero points, therefore maximum score for this task was 48. The test started with a tutorial with visual and auditory instructions, and two practice items that could be repeated. Instructions reminded participants to respond as quickly and accurately as possible. After the 24^th^ trial participants were presented with a screen that gave the option either to continue with the test or to take a break. The program recorded accuracy and response reaction time.

**Understanding Numbers** - Assessed mathematical achievement according to the standards of the UK National Curriculum. Items of this task were problems selected from the *Understanding* *Numbers* component of the nferNelson booklets (level 1 to 8) ([nferNelson, 1994](#_ENREF_12), [1999](#_ENREF_13), [2001](#_ENREF_14)). The solution of the problems requires understanding of the relationship between numerical expressions and patterns of numbers, understanding of mathematical operations, as well as of relationships among mathematical operations (e.g. division is the inverse of multiplication; this is one example of a trial: "Work out the value of x: 6x + 9 = 8x. Click on your answer" - 5 options were given as possible responses.). This test included both branching and discontinue rules. The 18 test items were arranged in increasing level of difficulty. The level of difficulty of each question was decided on the National Curriculum standardisation sample (reported in the Group Record Sheets; nferNelson 1994, 1999, 2001). Some of the questions used in the 16-year assessment were used for the mathematics web assessment at age 12. Difficulty level for such questions considered the previous TEDS assessment data as well as the nferNelson standardised data. The 18 questions were organized in 3 levels of 6 items each. Within each level the items were further divided into 3 sub-levels with increasing difficulty. All participants started with the same question of medium difficulty. The subsequent presentation order was determined by participants’ answers: answering correctly to the problems of one level advanced the test progressively to the more difficult questions; and items from the easier levels were credited as correct. If the problems within a level were answered incorrectly the test branched down to easier levels; the task stopped when three consecutive questions were answered incorrectly. The test started with a set of instructions and there was no practice trial. For some problems, the answers needed to be typed in, others had multiple choice answers and response required to click on the correct answers. For some problems, a simple calculator appeared on the screen alongside the question. After response was given, participants submitted their answers by clicking on the provided "ok" button. A new screen presented the option to progress to the next question or take a break and resume the test later. Maximum response time was 5 minutes and prompts encouraged participants to answer during this time. If no answer was given during the 5 minutes, participants were given the choice to go to the next question or take a break from the test. One point was awarded for each correct/credited answer; no points were given for timed out or incorrect answers, therefore maximum score on this test was 18. The program recorded accuracy and response reaction time.

**Corsi Tapping Block** - Assessed visuo-spatial working memory; it was programmed and adapted for on-line administration from the pen and paper version described in Farrell Pagulayan, Busch, Medina, Bartok and Krikorian ([2006](#_ENREF_3)). The number of items per trial was reduced from this version based on an internal validity analysis conducted on a pilot testing described in the following section. An image, depicting a black rectangle with 9 small cubes arranged inside was shown on the screen. The cubes lit up turning yellow for 1 sec in a patterned sequence, with a 1 sec interval between each cube. Participants had to reproduce the sequence by clicking on the cubes with a mouse. Trials were divided into 6 levels of difficulty - determined by the number of cubes lighting up, with two sequences within each level. To make the test age appropriate to our sample, the test started with 4 cubes lighting up in each sequence. In the hardest level 9 cubes lit up in each sequence. Immediately after the last cube lit up, a text prompt appeared on the screen inviting participants to start reproducing the sequence; they responded by clicking on the blocks in turn, using a mouse. As participants clicked on each block, this turned yellow and remained yellow until the next block was clicked. After each response participants were presented with the option to either continue with the test, or to come back to it later. The test had audio-visual instructions and 3-item practice trial which could be repeated until the participant was familiar with the task. If students correctly completed one or both sequences in a level, they progressed to the first item of the next level. The test was discontinued when both sequences in the same level were reproduced incorrectly. One point was assigned for each sequence correctly reproduced, with maximum score of 12. There was no time limit for response. The program recorded accuracy and reaction time for each trial.

**Reaction Time** - The task assessing processing speed measured response reaction time. It was programmed following the procedure described in Deary, Der and Ford ([2001](#_ENREF_2)). The numbers 1, 2, 3, 4 appeared 10 times each in a randomised order with a random interval between 1 and 3 seconds. Participants had to press the key corresponding to the number on the screen as quickly as possible. Presentation of the stimuli was in the same randomised order for all participants. The interval of 1 second between presentations was repeated 14 times and the interval of 2 and 3 seconds was repeated 13 times each. The task started with a 6-item practice trial. The practice trial could be repeated. Instructions reminded participants to respond as quickly and accurately as possible. Time out for responses was 8 seconds. If no response was given during this time the next trial followed and the question was recorded as incorrect. One point was assigned for each correct response for a maximum score of 40. The program recorded accuracy and response reaction time.

**Non-Verbal Ability** *- Raven Progressive Matrices* - This computerized test of non-verbal (fluid) intelligence was adapted from Raven, Court and Raven ([1996](#_ENREF_18)). Participants were presented with a matrix of patterns with one piece missing from each pattern. The task required to select the missing pattern from a choice of 8 by clicking on it with a mouse. The test consisted of 30 items organized in 3 levels with 6 items each, and a 4^th^ level with 12 items. There was a set of animated instructions and one practice trial that could be repeated at the discretion of the participant. The first 3 items of the first level were presented sequentially. Participants progressed within the same level if a correct response was given to at least one of the 3 items. If the first 3 items of the level were answered incorrectly, the following 3 items were skipped and the test advanced to the next level. One point was assigned for each correct answer; the skipped items received no points. The maximum score for this test was 30. If no answer was given within 5 minutes the program returned to the main page of the website. When resuming the session, the same question was presented. After each response, the next question followed immediately. Participants could, however, take a break at any point in the test. Accuracy and response reaction time was recorded by the program.

**Verbal Ability** - The test was programmed based on the Mill Hill Vocabulary test ([Raven, Raven, & Court, 1998](#_ENREF_17)). It consisted of 33 questions, where a single target-word was displayed on the screen with 6 other words below it. Target-words and the 6 responses were taken from the published measure. This is an example of target word: "fascinated" and the 6 choices: "ill-treated, poisoned, frightened, modelled, charmed, copied". The task required to click with a mouse on one of the words with the meaning closest to the target word presented on top. Only one of the 6 choices was the correct answer and one point score was assigned for each correct response. Maximum score for the test was 33. A set of instructions started the test followed by 1-item practice trial. A button to start the test appeared after the practice and the items were presented in the same order for all participants. After a response was given, the next question was presented immediately and it was possible to pause the test and resume it later. There was no time out for response. The test was discontinued after 7 consecutive items were answered incorrectly. Accuracy and response reaction time were recorded by the program.

**Language Ability** - was measured using the semantics *Figurative Language* subtest from the Test of Language Competences ([Wiig, Secord, & Sabers, 1989](#_ENREF_25)). The test was administered with the additional auditory modality to ensure that participants with reading problems would not be disadvantaged. The stimuli consisted of 15 target expressions or figures of speech, referred to a situation and were displayed one at a time with 4 other expressions having similar meaning. For example, the situation was described as "Two boys talking at a dog show"; the expression referred to this situation was: "He is crazy about that pet"; the 4 possible responses were: 1) "The pet makes him angry", 2) "He is up in arms about the pet", 3) “The pet is really wild", 4) "He is wild about the pet". The task required to match one of the 4 choices with the target expression. One point was assigned for each correct answer. A tutorial played at the beginning of the test advised to switch on the sound on the computer. There was one practice trial that provided feedback on response. During the practice and the test trials, the situation, target expression and the 4 choices were audio-played while the text was displayed on screen. It was possible to respond by clicking on the choice before the end of the audio recording. The next item was displayed immediately following the response. The choice to pause the test and resume it later was displayed following each item. Response time out was 60 sec. If during this time no answer was given, the question was recorded as incorrect and the next item was displayed. Accuracy and response reaction time were recorded by the program.

Reading was assessed with one measure of reading comprehension and one of reading fluency.

**Reading Comprehension** - The test was based on two passages of written text developed by Hayiou-Thomas & Dale (available from the authors). The task entailed reading the text and answering multiple choice comprehension questions based on the passage. Response was given by clicking on 1 of the 4 choices, of which, only one was correct. One point score was assigned for each correct answer. Thirteen questions were asked for each of the two passages, with maximum total score of 26. The test started with an introduction tutorial and there was no practice. After the tutorial, the first passage was presented on the screen, with an option to click on the “next” button to proceed to the first question and multiple choice answers. The text remained on the screen together with the questions. The next question appeared immediately following the response. It was possible to pause the test at any time. Time-out for each response was 5 minutes. If during this time no answer was given, the question was recorded as wrong and the next question followed. The reading time was recorded from the appearance of the passage to the click of the “next” button to see the first question. The program recorded accuracy and response reaction time.

**Reading Fluency** - The test was programmed according to Woodcock-Johnson III ([Woodcock, McGrew, & Mather, 2001](#_ENREF_26)). Participants had 2 minutes and 30 seconds to respond to as many as of the 98 statements which required a yes/no answer (for example “A jug may be used to pour water”). Response was given by clicking with a mouse on the “Yes” or “No” buttons displayed together with each statement. The next item was displayed immediately following the response. One point was awarded for each correct answer; no points were assigned for timed out or incorrect responses. Maximum time for a response was 40 seconds, if no response was given during this time the question was recorded as incorrect and the next question was presented. The test was provided with a set of instructions and one practice trial with feedback. The program recorded accuracy and response reaction time. The total time of completion was also recorded.

**Validation**

**Pilot of the measures** *-* The tests used to measure language, reading, verbal and non-verbal abilities at 16 were adapted from previous versions of the TEDS web batteries. Validation and reliability of these measures can be found in previous TEDS publications ([e.g. Haworth et al., 2007](#_ENREF_7)). The five new tests administered at age 16 (Corsi, Reaction Time, Problem Verification, Number Line and Dot Task) were piloted and tested for reliability and suitability for web administration using a sample of 100 16 years old singleton students (Tosto, Tikhomirova, Galajinsky, Akimova, & Kovas, 2013). In the first phase of the pilot, students were individually assessed; Corsi and Number Line tests were administered in a pen and paper format, Reaction Time, Problem Verification and Dot Task were administered using a laptop. Based on this first wave of assessment, some of the tasks were modified for web implementation. During the second phase of the pilot, five months later, all measures were administered to the same participants online. All tests were successfully completed on the web, and showed a good internal validity in both phases of the pilot with Cronbach alpha values ranging between .75 (Number Line and Corsi) and .94 (Reaction Time accuracy and response time). Because of the modifications to most of the tasks, as well as the change in administration format between the first and the second phase of the pilot, the test-retest correlations were reduced and therefore underestimate reliability. For the least modified Corsi and Problem Verification tests these were .5 and .6 respectively.

**TEDS tests re-test** - About two months after the start of TEDS' web assessment at age 16, 24 pairs of twins were invited to the SGDP (Social, Genetic and Developmental Psychiatry) Centre King's College London, to repeat all the activities for test-retest reliability purposes. By that date, about 600 families had completed the online battery. This validation sample was selected based on the data from the “g” tests (Mill Hill Vocabulary and Raven’s Matrices) and the socio-economic status (SES) composite obtained from the parents’ web questionnaire, as part of the online parents' consent form. The mean “g” and SES of the validation sample was matched to the “g” and SES averages of the unselected twins. Test re-test correlations are reported in Table 2 of the main manuscript.

**Measures age 7 to 14**

Full description and validation of the measures at these ages can be found in previous TEDS publications (e.g. Haworth et al., 2007; Kovas, Haworth, Dale, & Plomin, 2007). A brief description is provided below.

**7 years.** Data at 7 were collected using telephone testing for the cognitive abilities and teacher questionnaire for mathematical school achievement. *Verbal Ability* was a composite measure obtained from the WISC-III “Vocabulary” and “Similarities” sub tests ([Wechsler, 1992](#_ENREF_24)). *Non-Verbal Ability* was a composite score of the “Conceptual Grouping” test ([McCarthy, 1972](#_ENREF_10)) and WISC-III “Picture Completion” test (Wechsler, 1992). *Reading* *Ability* was measured by the TOWRE which assesses fluency of reading words and non-words ([Torgesen, Wagner, & Rashotte, 1999](#_ENREF_22)). *Mathematics Teachers’ Ratings* was a composite score derived from the teacher ratings of children’s achievement, based on the expected UK standard at Key stage 1 developed by the Qualifications and Curriculum Authority (QCA: www.qca.org.uk) on three mathematical components: using and applying mathematics; numbers; shapes, space and measures.

**9 years.** Data were collected by postal booklets. The cognitive measures were derived from the child-completed booklets. *Verbal Ability* score was obtained combining the scores of “Vocabulary Multiple Choice” and “General Knowledge” tests taken from WISC-III-PI ([Kaplan, Fein, Kramer, Delis, & Morris, 1999](#_ENREF_8)). *Non Verbal Ability* was a composite of the scores of the “Puzzle” and “Shapes” tests adapted from Smith, Fernandes and Strand ([2001](#_ENREF_20)). *Mathematics Teachers’ Ratings* was a composite score of teacher ratings of the twins' academic achievement based on the UK standard at Key stage 2 of the Qualifications and Curriculum Authority (QCA: www.qca.org.uk) on three mathematical components: using and applying mathematics; numbers and algebra; shapes, space and measures.

**10 years.** Data were collected using a web-based test battery. *Verbal Ability* was obtained by combining the “Vocabulary Multiple Choice” and “General Knowledge” tests from the WISC-III-PI ([Kaplan, et al., 1999](#_ENREF_8)). A *Non Verbal Ability* scale was obtained from two web tests: “Picture Completion” ([Wechsler, 1992](#_ENREF_24)) and the “Raven Standard Progressive Matrices” ([Raven et al., 1996](#_ENREF_18)). *Reading Ability* was assessed with the test of reading comprehension PIAT ([Peabody Individual Achievement Test; Markwardt 1997](#_ENREF_9)). *Mathematics Web Test* was based on the items taken from the NFER 5–14 Mathematics Series that assessed 3 mathematical sub-components: non-numerical processes, understanding numbers, computation and knowledge. In addition to mathematical web assessment, three mathematics components of: using and applying mathematics, number and algebra, shapes, space and measures, were assessed by teacher questionnaires based on the UK National Curriculum standards at Key Stage 2 of the Qualifications and Curriculum Authority (QCA, 2001: http://curriculum.qca.org.uk/) and were combined into a *Mathematics Teachers’ Ratings* score.

**12 years.** Data were collected using a web-based test battery. A measure of *Verbal Ability* combined the scores of “Vocabulary Multiple Choice” and “General Knowledge” tests from the WISC-III- PI ([Kaplan, et al., 1999](#_ENREF_8)). *Non Verbal Ability* composite score was obtained from “Raven’s Standard Progressive Matrices” ([Raven et al., 1996](#_ENREF_18)) and the “Picture Completion” test ([Wechsler, 1992](#_ENREF_24)) tests. *Reading Ability* composite score was obtained from 3 tests of reading fluency and 2 tests of reading comprehension: reading words and non words subtests of the reading fluency test ([Torgesen, et al., 1999](#_ENREF_22)); Woodcock-Johnson III Reading Fluency Test ([Woodcock, et al., 2001](#_ENREF_26)); GOAL, reading comprehension ([GOAL plc, 2002](#_ENREF_4)); PIAT, reading comprehension (Markwardt, 1997). *Language Ability* composite score was obtained from 3 tests: “Figurative Language”, the test of semantic language competence (Wiig et al., 1989); “Inferences”, the test of pragmatics skills (Wiig et al., 1989); and TOAL-3, the test of grammar ([Hammill, Brown, Larsen, & Wiederholt, 1994](#_ENREF_6)). *Spatial Ability* composite score was obtained from the “Hidden Shapes” and the “Jigsaw” tests from the nferNelson Spatial Reasoning Series booklets ([Smith & Lord, 2002](#_ENREF_21)). *Mathematics Web test* was based on nferNelson 5–14 Mathematics Series, assessing 3 mathematical components: understanding numbers, non-numerical processes, computation and knowledge ([nferNelson, 1994](#_ENREF_12), [1999](#_ENREF_13), [2001](#_ENREF_14)). In addition, *Mathematics Teachers’ Ratings* was assessed by teachers on 4 mathematical components: using and applying mathematics; numbers and algebra; shape space and measures; handling data. Ratings were based on the UK National Curriculum of the Qualifications and Curriculum Authority (QCA, 2001: http://curriculum.qca.org.uk/).

**14 years.** Data for cognitive tests were collected using a web-based test battery. *Verbal Ability* was assessed with the “Vocabulary Multiple Choice” from the WISC-III- PI ([Kaplan, et al., 1999](#_ENREF_8)). *Non Verbal Ability* was measured by the “Raven’s Standard Progressive Matrices” test ([Raven et al., 1996](#_ENREF_18)). *Mathematics Teachers’ Ratings* was assessed by teachers’ ratings of mathematics: using and applying mathematics; numbers and algebra; shape space and measures; handling data - based on the UK National Curriculum of the Qualifications and Curriculum Authority (QCA, 2001: http://curriculum.qca.org.uk/).

**Relationship between the two estimation measures**

One of the aims of the study was to examine the extent to which symbolic and non-symbolic estimation processes are related to each other at the age of 16. Number line and dot estimation correlated with each other modestly, r = .22, 95% CI [.18; .26]. We further explored the construct of estimation at age 16 by entering number line and dot estimation into exploratory Principal Axis Factoring Analysis (PAF) together with other cognitive measures collected at this age. Analyses were performed with both orthogonal (Varimax) and oblique (Oblimin) rotations. The adequacy of the sample size (1835 participants with complete data) was confirmed with Kaiser Meyer Olkin (KMO) test which returned a good value of .87, with the Bartlett’s sphericity test χ^2^ (45) = 5223.23, p < .001. By choosing the number of factors based on eigenvalues > 1, both rotations generated models with two factors (confirmed by the scree plot). Factors generated with orthogonal rotation showed cross-loadings from language, reading, mathematics (GCSE and web scores), non verbal ability and speed of processing. Symbolic and non-symbolic estimation loaded on the same factor. With the oblique rotation, only mathematics GCSE loaded on both factors; in this model, the two factors identified what can be considered a non-verbal dimension, which included symbolic and non-symbolic estimation, mathematics, non verbal ability, speed of processing and memory scores (variance explained 34.04%, eigenvalue = 3.91). Reading, language, verbal ability and GCSE loaded on a second factor (variance explained 4.93%, eigenvalue = 1.11). Details of the factors and their loadings in the two models can be found in Table S2.

As initial extractions identified a third factor with an eigenvalue of .91 we further explored our measures, with a more flexible 3-factors model. We performed two more PAFs with orthogonal and oblique rotation requiring the extraction of three factors. The orthogonal rotation generated factors with small loadings from almost all the variables. This indicates that variances could not be kept independent by the orthogonality of the factors. The oblique rotation presented a cleaner output in terms of factor loadings (Table S2).

| Table S2 | | | | | | | | | | | | | | |
| --- | --- | --- | --- | --- | --- | --- | --- | --- | --- | --- | --- | --- | --- | --- |
| *Exploratory Factor Analysis* | | | | | | | | | | | | | | |
|  | Extraction method: Kaiser criterion,eigen values >1 | | | | | |  | Extraction method: 3 factors required | | | | | | |
|  | Orthogonal rotation (Varimax) | | |  | Oblique rotation (Oblimin) | |  | Orthogonal rotation (Varimax) | | |  | Oblique rotation (Oblimin) | | |
|  |  | | |  |  | |  |  | | |  |  | | |
|  | Total Variance explained after rotation 38.98% | | |  | Total Variance explained after rotation 38.98% | |  | Total Variance explained after rotation 41.96% | | |  | Total Variance explained after rotation 41.96% | | |
|  |  | | |  |  | |  |  | | |  |  | | |
|  | Factor 1 | Factor 2 | |  | Factor 1 | Factor 2 |  | Factor 1 | Factor 2 | Factor 3 |  | Factor 1 | Factor 2 | Factor 3 |
| Language | .29 | .63 | |  |  | .62 |  | .27 | .61 |  |  |  | .60 |  |
| Reading | .24 | .72 | |  |  | .76 |  |  | .72 | .22 |  |  | .76 |  |
| Verbal ability |  | .59 | |  |  | .63 |  |  | .58 |  |  |  | .60 |  |
| GSCEs Maths | .64 | .47 | |  | .60 | .27 |  | .66 | .45 |  |  | .73 | .23 |  |
| Web Maths | .79 | .37 | |  | .82 |  |  | .80 | .33 | .25 |  | .94 |  |  |
| Non verbal Ability (Raven) | .52 | .34 | |  | .51 |  |  | .46 | .32 | .26 |  | .46 |  |  |
| Memory (Corsi) | .39 |  | |  | .44 |  |  | .31 |  | .25 |  | .33 |  |  |
| Speed of processing (Reaction time) | -.33 | -.25 | |  | -.30 |  |  |  | -.22 | -.41 |  |  |  | .37 |
| Number Line estimation | -.46 |  | |  | -.52 |  |  | -.37 |  | -.27 |  | -.40 |  |  |
| Dot estimation | -.33 |  | |  | -.35 |  |  |  |  | -.46 |  |  |  | .45 |
| % variance explained | *34.04* | *4.93* | |  | *34.04* | *4.93* |  | *34.32* | *5.10* | *2.53* |  | *34.33* | *5.11* | *2.53* |
| Eigen values | *3.91* | *1.11* | |  | *3.91* | *1.11* |  | *3.91* | *1.11* | *.91* |  | *3.91* | *1.11* | *.91* |
| α | *.42* | | *.72* |  | *.10* | *.78* |  | *.56* | *.72* | *.02* |  | *.45* | *.78* | *.42* |
| Note: Principal Axis Factoring Analysis conducted on cognitive and achievement measures collected at age 16. | | | | | | | | | | | | | | |

Number line estimation loaded onto a non verbal factor (variance explained 34.33%, eigenvalue = 3.91) together with mathematics (GCSE and web scores), non verbal ability and memory scores. Mathematics loaded strongly on this factor (average loading of GCSE and web scores was .84), while number line estimation, memory and non verbal scores had similar loadings (average .43). The second factor (variance explained 5, 11%, eigenvalue = 1.11) identified a verbal dimension; reading, language and verbal ability scores loaded strongly on it (average .66) and there was a small loading (.23) of mathematics GCSE scores. Dot estimation loaded onto a third factor (eigenvalue = .91), together with speed of processing scores; this factor explained 2.53% of the variance in the model. These results show that even allowing separation from the other variables, number line and dot estimation did not cluster together, suggesting heterogeneity within the estimation domain.

**References**

Booth, J. L., & Siegler, R. S. (2006). Developmental and individual differences in pure numerical estimation. *Developmental Psychology, 42*(1), 189.

Deary, I. J., Der, G., & Ford, G. (2001). Reaction times and intelligence differences: A population-based cohort study. *Intelligence, 29*(5), 389-399.

Farrell Pagulayan, K., Busch, R. M., Medina, K. L., Bartok, J. A., & Krikorian, R. (2006). Developmental normative data for the Corsi Block-tapping task. *Journal of Clinical and Experimental Neuropsychology, 28*(6), 1043-1052.

GOAL. (2002). *GOAL Formative Assessment: Key Stage 3*. London: Hodder & Stoughton.

Halberda, J., Mazzocco, M. M. M., & Feigenson, L. (2008). Individual differences in non-verbal number acuity correlate with maths achievement. *Nature, 455*(7213), 665-668.

Hammill, D. D., Brown, V. L., Larsen, S. C., & Wiederholt, J. L. (1994). Test of adolescent and adult language. Austin, Texas: Pro-Ed

Haworth, C. M., Harlaar, N., Kovas, Y., Davis, O. S., Oliver, B. R., Hayiou-Thomas, M. E., . . . Dale, P. S. (2007). Internet cognitive testing of large samples needed in genetic research. *Twin Research and Human Genetics, 10*(4), 554-563.

Kaplan, E., Fein, D., Kramer, J., Delis, D., & Morris, R. (1999). The WISC-III as a process instrument. *San Antonio, Texas: The Psychological Corporation*.

Markwardt , F. C. (1997). *Peabody individual achievement test-revised (normative update) manual*. Cirle Pines, MN: American Guidance Service.

McCarthy, D. (1972). *Manual for the McCarthy scales of children's abilities*. New York: Psychological Corporation.

Murphy, M. M., & Mazzocco, M. M. (2008). Mathematics learning disabilities in girls with fragile X or Turner syndrome during late elementary school. *Journal of Learning Disabilities, 41*(1), 29-46.

nferNelson. (1994). *Maths 5–14 series*. London: nferNelson Publishing Company Ltd.

nferNelson. (1999). *Maths 5–14 series*. London: nferNelson Publishing Company Ltd.

nferNelson. (2001). *Maths 5–14 series*. London: nferNelson Publishing Company Ltd.

Nys, J., & Content, A. (2012). Judgement of discrete and continuous quantity in adults: number counts! *The Quarterly Journal of Experimental Psychology, 65*(4), 675-690.

Opfer, J. E., & Siegler, R. S. (2007). Representational change and children’s numerical estimation. *Cognitive Psychology, 55*(3), 169-195.

Raven, J., Raven, J., C,, & Court, J. H. (1998). *Mill Hill vocabulary scale.* Oxford: Oxford Psychologists Press.

Raven, J. C., Court, J. H., & Raven, J. (1996). *Manual for Raven's Progressive Matrices and Vocabulary Scales*. Oxford: Oxford University Press.

Siegler, R. S., & Booth, J. L. (2004). Development of numerical estimation in young children. *Child Development, 75*(2), 428-444.

Smith, P., Fernandes, C., & Strand, S. (2001). *Cognitive Abilities Test 3 (CAT3)*. Windsor, England: nferNelson.

Smith, P., & Lord, T. (2002). *Spatial reasoning 6-14 series, a teacher's guide*. London, UK: nferNelson Publishing Company Ltd.

Torgesen, J. K., Wagner, R. K., & Rashotte, C. A. (1999). Test of word reading efficiency (TOWRE). *Austin, Texas: Pro-Ed*.

Van Selst, M., & Jolicoeur, P. (1994). A solution to the effect of sample size on outlier elimination. *The Quarterly Journal of Experimental Psychology, 47*(3), 631-650.

Wechsler, D. (1992). *Wechsler intelligence scale for children* (3rd ed.). UK: The Psychological Corporation.

Wiig, E. H., Secord, W., & Sabers, D. (1989). *Test of Language Competence: Expanded Edition*. San Antonio, TX: Psychological Corporation.

Woodcock, R., McGrew, K., & Mather, N. (2001). *Woodcock–Johnson III (WJ-III)*. Itasca,

| Table S3 | | | | | | | | | | | | |
| --- | --- | --- | --- | --- | --- | --- | --- | --- | --- | --- | --- | --- |
| *Descriptive statistics measures from age 7 to 14 for all sample, males and females separately* | | | | | | | | | | | | |
|  | n All | Mean All | Median All | Std. Dev. All | n Males | Mean Males | Median Males | Std. Dev. Males | n Fem. | Mean Fem. | Median Fem. | Std. Dev. Fem. |
| Mathematics teacher at 7 | 6058 | .04 | -.12 | .93 | 2934 | .06 | -.11 | .97 | 3124 | .02 | -.14 | .88 |
| Verbal ability at 7 | 5173 | -.01 | .01 | .98 | 2498 | -.02 | .00 | 1.00 | 2675 | .00 | .02 | .96 |
| Non verbal ability at 7 | 5205 | .01 | .08 | .99 | 2519 | -.03 | .01 | 1.00 | 2686 | .05 | .12 | .98 |
| Reading at 7 | 5338 | .00 | -.01 | 1.00 | 2592 | -.05 | -.07 | 1.03 | 2746 | .05 | .04 | .97 |
|  |  |  |  |  |  |  |  |  |  |  |  |  |
| Mathematics teacher at 9 | 2683 | .00 | .03 | .97 | 1261 | .06 | .04 | 1.00 | 1422 | -.05 | .01 | .95 |
| Verbal ability at 9 | 3229 | .02 | .06 | .97 | 1496 | .05 | .06 | .96 | 1733 | -.01 | .05 | .98 |
| Non verbal ability at 9 | 3154 | .01 | .29 | .99 | 1458 | .02 | .31 | .99 | 1696 | .01 | .28 | .98 |
|  |  |  |  |  |  |  |  |  |  |  |  |  |
| Mathematics teacher at 10 | 2813 | .04 | -.22 | .96 | 1324 | .10 | .00 | .99 | 1489 | -.02 | -.30 | .93 |
| Mathematics web at 10 | 2827 | .04 | .26 | .93 | 1282 | .11 | .36 | .92 | 1545 | -.02 | .20 | .93 |
| Verbal ability at 10 | 2567 | .00 | .13 | .98 | 1135 | .14 | .29 | .96 | 1432 | -.10 | -.04 | .98 |
| Non verbal ability at 10 | 2562 | .02 | .12 | .95 | 1134 | .06 | .17 | .96 | 1428 | -.01 | .09 | .94 |
| Reading at 10 | 3088 | .01 | .05 | .98 | 1416 | .01 | .08 | 1.03 | 1672 | .00 | .03 | .94 |
|  |  |  |  |  |  |  |  |  |  |  |  |  |
| Mathematics teacher at 12 | 3727 | -.03 | .06 | .91 | 1745 | .00 | .10 | .93 | 1982 | -.05 | .03 | .89 |
| Mathematics web at 10 | 5178 | .03 | .20 | .94 | 2364 | .08 | .29 | .96 | 2813 | -.01 | .15 | .93 |
| Verbal ability at 12 | 4375 | .01 | .18 | .98 | 1937 | .13 | .30 | .96 | 2438 | -.08 | .06 | .99 |
| Non verbal ability at 12 | 4230 | .02 | .06 | .96 | 1870 | .03 | .09 | 1.00 | 2360 | .01 | .04 | .94 |
| Spatial ability at 12 | 5156 | .00 | -.04 | .97 | 2319 | .04 | .03 | 1.01 | 2836 | -.03 | -.09 | .95 |
| Reading at 12 | 5664 | .03 | .11 | .97 | 2610 | -.01 | .07 | .98 | 3053 | .05 | .13 | .96 |
| Language at 12 | 4687 | .01 | .10 | .99 | 2096 | -.01 | .08 | 1.00 | 2591 | .03 | .12 | .98 |
|  |  |  |  |  |  |  |  |  |  |  |  |  |
| Mathematics teacher at 14 | 466 | .04 | .05 | .98 | 207 | .06 | .05 | 1.02 | 259 | .02 | .05 | .96 |
| Verbal ability at 14 | 3303 | .05 | .14 | .95 | 1384 | .02 | .11 | .96 | 1919 | .07 | .17 | .95 |
| Non verbal ability 14 | 2802 | .00 | .02 | .97 | 1195 | .00 | .04 | 1.00 | 1607 | .01 | .02 | .96 |
| *Note.* n = sample size constituted by one randomly selected twin in each pair. Descriptive statistics are reported on variables standardized to a mean of 1.00 and standard deviation of .00, corrected for age, scores outside ± 3 standard deviation excluded. Fem. = Females. | | | | | | | | | | | | |
|  | | | | | | | | | | | | |

IL: Riverside.

| Table S4  *Correlations measures age 7 to 16* | | | | | | | | | | | | | | | | | | | | | | | | | | | | | | | | | |
| --- | --- | --- | --- | --- | --- | --- | --- | --- | --- | --- | --- | --- | --- | --- | --- | --- | --- | --- | --- | --- | --- | --- | --- | --- | --- | --- | --- | --- | --- | --- | --- | --- | --- |
|  |  | **1** | **2** | **3** | **4** | **5** | **6** | **7** | **8** | **9** | **10** | **11** | **12** | **13** | **14** | **15** | **16** | **17** | **18** | **19** | **20** | **21** | **22** | **23** | **24** | **25** | **26** | **27** | **28** | **29** | **30** | **31** | **32** |
| **1** | Number Line 16  n | 1 |  |  |  |  |  |  |  |  |  |  |  |  |  |  |  |  |  |  |  |  |  |  |  |  |  |  |  |  |  |  |  |
|  |  | 2792 |  |  |  |  |  |  |  |  |  |  |  |  |  |  |  |  |  |  |  |  |  |  |  |  |  |  |  |  |  |  |  |
| **2** | WebFract.16 n | .22^**^ | 1. |  |  |  |  |  |  |  |  |  |  |  |  |  |  |  |  |  |  |  |  |  |  |  |  |  |  |  |  |  |  |
|  |  | 2412 | 2437 |  |  |  |  |  |  |  |  |  |  |  |  |  |  |  |  |  |  |  |  |  |  |  |  |  |  |  |  |  |  |
| **3** | Maths Teach. 7  n | -.28^**^ | -.23^**^ | 1. |  |  |  |  |  |  |  |  |  |  |  |  |  |  |  |  |  |  |  |  |  |  |  |  |  |  |  |  |  |
|  |  | 1893 | 1674 | 6058 |  |  |  |  |  |  |  |  |  |  |  |  |  |  |  |  |  |  |  |  |  |  |  |  |  |  |  |  |  |
| **4** | Verbal 7  n | -.15^**^ | -.11^**^ | .39^**^ | 1. |  |  |  |  |  |  |  |  |  |  |  |  |  |  |  |  |  |  |  |  |  |  |  |  |  |  |  |  |
|  |  | 2220 | 1953 | 3931 | 5173 |  |  |  |  |  |  |  |  |  |  |  |  |  |  |  |  |  |  |  |  |  |  |  |  |  |  |  |  |
| **5** | Non-Verbal 7 n | -.14^**^ | -.12^**^ | .27^**^ | .39^**^ | 1. |  |  |  |  |  |  |  |  |  |  |  |  |  |  |  |  |  |  |  |  |  |  |  |  |  |  |  |
|  |  | 2235 | 1965 | 3955 | 5151 | 5205 |  |  |  |  |  |  |  |  |  |  |  |  |  |  |  |  |  |  |  |  |  |  |  |  |  |  |  |
| **6** | Reading 7  n | -.22^**^ | -.15^**^ | .54^**^ | .42^**^ | .27^**^ | 1 |  |  |  |  |  |  |  |  |  |  |  |  |  |  |  |  |  |  |  |  |  |  |  |  |  |  |
|  |  | 2252 | 1980 | 4007 | 5095 | 5121 | 5338 |  |  |  |  |  |  |  |  |  |  |  |  |  |  |  |  |  |  |  |  |  |  |  |  |  |  |
| **7** | Maths Teach. 9  n | -.30^**^ | -.21^**^ | .59^**^ | .38^**^ | .30^**^ | .54^**^ | 1 |  |  |  |  |  |  |  |  |  |  |  |  |  |  |  |  |  |  |  |  |  |  |  |  |  |
|  |  | 1581 | 1397 | 2004 | 2280 | 2300 | 2309 | 2683 |  |  |  |  |  |  |  |  |  |  |  |  |  |  |  |  |  |  |  |  |  |  |  |  |  |
| **8** | Verbal 9  n | -.15^**^ | -.07^**^ | .27^**^ | .34^**^ | .24^**^ | .33^**^ | .32^**^ | 1 |  |  |  |  |  |  |  |  |  |  |  |  |  |  |  |  |  |  |  |  |  |  |  |  |
|  |  | 2023 | 1802 | 2400 | 2786 | 2803 | 2823 | 2274 | 3229 |  |  |  |  |  |  |  |  |  |  |  |  |  |  |  |  |  |  |  |  |  |  |  |  |
| **9** | Non-Verbal 9  n | -.23^**^ | -.18^**^ | .37^**^ | .33^**^ | .27^**^ | .30^**^ | .39^**^ | .41^**^ | 1 |  |  |  |  |  |  |  |  |  |  |  |  |  |  |  |  |  |  |  |  |  |  |  |
|  |  | 1989 | 1779 | 2352 | 2718 | 2736 | 2754 | 2219 | 3140 | 3154 |  |  |  |  |  |  |  |  |  |  |  |  |  |  |  |  |  |  |  |  |  |  |  |
| **10** | Maths Teach. 10  n | -.31^**^ | -.17^**^ | .55^**^ | .38^**^ | .28^**^ | .53^**^ | .63^**^ | .32^**^ | .38^**^ | 1 |  |  |  |  |  |  |  |  |  |  |  |  |  |  |  |  |  |  |  |  |  |  |
|  |  | 1694 | 1491 | 2103 | 2383 | 2397 | 2420 | 1953 | 2301 | 2257 | 2813 |  |  |  |  |  |  |  |  |  |  |  |  |  |  |  |  |  |  |  |  |  |  |
| **11** | Maths Web 10  n | -.30^**^ | -.20^**^ | .44^**^ | .30^**^ | .27^**^ | .37^**^ | .47^**^ | .39^**^ | .47^**^ | .50^**^ | 1 |  |  |  |  |  |  |  |  |  |  |  |  |  |  |  |  |  |  |  |  |  |
|  |  | 1870 | 1675 | 2013 | 2342 | 2358 | 2382 | 1767 | 2261 | 2223 | 1965 | 2827 |  |  |  |  |  |  |  |  |  |  |  |  |  |  |  |  |  |  |  |  |  |
| **12** | Verbal 10  n | -.22^**^ | -.15^**^ | .34^**^ | .37^**^ | .24^**^ | .33^**^ | .37^**^ | .49^**^ | .37^**^ | .36^**^ | .53^**^ | 1 |  |  |  |  |  |  |  |  |  |  |  |  |  |  |  |  |  |  |  |  |
|  |  | 1741 | 1554 | 1864 | 2151 | 2168 | 2190 | 1698 | 2176 | 2141 | 1893 | 2551 | 2567 |  |  |  |  |  |  |  |  |  |  |  |  |  |  |  |  |  |  |  |  |
| **13** | Non-Verbal 10  n | -.25^**^ | -.20^**^ | .32^**^ | .26^**^ | .29^**^ | .21^**^ | .35^**^ | .32^**^ | .44^**^ | .34^**^ | .56^**^ | .54^**^ | 1 |  |  |  |  |  |  |  |  |  |  |  |  |  |  |  |  |  |  |  |
|  |  | 1735 | 1551 | 1861 | 2148 | 2165 | 2187 | 1692 | 2173 | 2138 | 1889 | 2548 | 2554 | 2562 |  |  |  |  |  |  |  |  |  |  |  |  |  |  |  |  |  |  |  |
| **14** | Reading 10  n | -.22^**^ | -.16^**^ | .34^**^ | .36^**^ | .25^**^ | .44^**^ | .38^**^ | .42^**^ | .34^**^ | .36^**^ | .51^**^ | .53^**^ | .46^**^ | 1 |  |  |  |  |  |  |  |  |  |  |  |  |  |  |  |  |  |  |
|  |  | 1985 | 1763 | 2176 | 2534 | 2550 | 2578 | 1906 | 2422 | 2378 | 2118 | 2822 | 2563 | 2558 | 3088 |  |  |  |  |  |  |  |  |  |  |  |  |  |  |  |  |  |  |
| **15** | Maths teach. 12  n | -.33^**^ | -.20^**^ | .56^**^ | .37^**^ | .27^**^ | .48^**^ | .53^**^ | .31^**^ | .40^**^ | .53^**^ | .50^**^ | .37^**^ | .32^**^ | .34^**^ | 1 |  |  |  |  |  |  |  |  |  |  |  |  |  |  |  |  |  |
|  |  | 1014 | 907 | 2686 | 1912 | 1920 | 1939 | 954 | 1206 | 1175 | 1031 | 967 | 935 | 937 | 1045 | 3727 |  |  |  |  |  |  |  |  |  |  |  |  |  |  |  |  |  |
| **16** | Maths Web 12  n | -.36^**^ | -.27^**^ | .49^**^ | .35^**^ | .29^**^ | .42^**^ | .53^**^ | .37^**^ | .49^**^ | .53^**^ | .61^**^ | .45^**^ | .48^**^ | .45^**^ | .55^**^ | 1 |  |  |  |  |  |  |  |  |  |  |  |  |  |  |  |  |
|  |  | 2018 | 1789 | 3683 | 2914 | 2935 | 2953 | 1698 | 2196 | 2158 | 1863 | 2036 | 1929 | 1925 | 2142 | 2771 | 5178 |  |  |  |  |  |  |  |  |  |  |  |  |  |  |  |  |
| **17** | Verbal 12  n | -.25^**^ | -.17^**^ | .37^**^ | .43^**^ | .27^**^ | .36^**^ | .43^**^ | .47^**^ | .38^**^ | .41^**^ | .47^**^ | .56^**^ | .40^**^ | .48^**^ | .41^**^ | .52^**^ | 1 |  |  |  |  |  |  |  |  |  |  |  |  |  |  |  |
|  |  | 1766 | 1571 | 3146 | 2461 | 2476 | 2494 | 1483 | 1960 | 1920 | 1621 | 1798 | 1743 | 1742 | 1880 | 2492 | 4084 | 4375 |  |  |  |  |  |  |  |  |  |  |  |  |  |  |  |
| **18** | Non verbal 12  n | -.26^**^ | -.21^**^ | .30^**^ | .28^**^ | .30^**^ | .24^**^ | .32^**^ | .27^**^ | .44^**^ | .32^**^ | .40^**^ | .36^**^ | .55^**^ | .36^**^ | .35^**^ | .53^**^ | .45^**^ | 1 |  |  |  |  |  |  |  |  |  |  |  |  |  |  |
|  |  | 1723 | 1536 | 3043 | 2367 | 2384 | 2399 | 1437 | 1898 | 1861 | 1572 | 1739 | 1687 | 1687 | 1815 | 2427 | 3989 | 4223 | 4230 |  |  |  |  |  |  |  |  |  |  |  |  |  |  |
| **19** | Reading 12  n | -.25^**^ | -.19^**^ | .47^**^ | .44^**^ | .28^**^ | .67^**^ | .49^**^ | .40^**^ | .33^**^ | .50^**^ | .43^**^ | .44^**^ | .31^**^ | .55^**^ | .47^**^ | .56^**^ | .52^**^ | .37^**^ | 1 |  |  |  |  |  |  |  |  |  |  |  |  |  |
|  |  | 2188 | 1939 | 3999 | 3234 | 3259 | 3287 | 1874 | 2423 | 2381 | 2046 | 2237 | 2111 | 2108 | 2370 | 2983 | 5159 | 4283 | 4179 | 5664 |  |  |  |  |  |  |  |  |  |  |  |  |  |
| **20** | Language 12  n | -.23^**^ | -.17^**^ | .39^**^ | .46^**^ | .28^**^ | .374^**^ | .44^**^ | .39^**^ | .42^**^ | .424^**^ | .46^**^ | .46^**^ | .38^**^ | .48^**^ | .43^**^ | .56^**^ | .60^**^ | .48^**^ | .56^**^ | 1 |  |  |  |  |  |  |  |  |  |  |  |  |
|  |  | 1869 | 1660 | 3355 | 2639 | 2657 | 2677 | 1538 | 2027 | 1989 | 1681 | 1912 | 1804 | 1802 | 2004 | 2564 | 4361 | 4369 | 4227 | 4572 | 4687 |  |  |  |  |  |  |  |  |  |  |  |  |
| **21** | Spatial 12  n | -.28^**^ | -.19^**^ | .26^**^ | .19^**^ | .22^**^ | .17^**^ | .25^**^ | .22^**^ | .34^**^ | .28^**^ | .32^**^ | .25^**^ | .39^**^ | .27^**^ | .29^**^ | .46^**^ | .32^**^ | .47^**^ | .28^**^ | .35^**^ | 1 |  |  |  |  |  |  |  |  |  |  |  |
|  |  | 2070 | 1838 | 3648 | 2978 | 2999 | 3025 | 1737 | 2275 | 2239 | 1889 | 2104 | 1985 | 1981 | 2223 | 2741 | 4722 | 4287 | 4214 | 5084 | 4567 | 5156 |  |  |  |  |  |  |  |  |  |  |  |
| **22** | Maths teach. 14  n | -.40^**^ | -.22^**^ | .57^**^ | .39^**^ | .39^**^ | .50^**^ | .60^**^ | .38^**^ | .42^**^ | .64^**^ | .54^**^ | .47^**^ | .42^**^ | .35^**^ | .61^**^ | .65^**^ | .47^**^ | .34^**^ | .46^**^ | .56^**^ | .40^**^ | 1 |  |  |  |  |  |  |  |  |  |  |
|  |  | 315 | 283 | 334 | 397 | 399 | 396 | 262 | 331 | 326 | 261 | 267 | 250 | 250 | 287 | 270 | 305 | 236 | 228 | 356 | 264 | 351 | 466 |  |  |  |  |  |  |  |  |  |  |
| **23** | Verbal 14  n | -.24^**^ | -.21^**^ | .34^**^ | .38^**^ | .21^**^ | .39^**^ | .38^**^ | .40^**^ | .36^**^ | .39^**^ | .37^**^ | .45^**^ | .30^**^ | .40^**^ | .41^**^ | .45^**^ | .52^**^ | .33^**^ | .50^**^ | .49^**^ | .28^**^ | .48^**^ | 1 |  |  |  |  |  |  |  |  |  |
|  |  | 1556 | 1400 | 2333 | 1974 | 1989 | 1995 | 1171 | 1549 | 1523 | 1238 | 1375 | 1309 | 1305 | 1463 | 1761 | 2670 | 2388 | 2330 | 2874 | 2502 | 2729 | 367 | 3303 |  |  |  |  |  |  |  |  |  |
| **24** | Non verbal 14  n | -.29^**^ | -.25^**^ | .37^**^ | .28^**^ | .24^**^ | .31^**^ | .36^**^ | .23^**^ | .39^**^ | .42^**^ | .41^**^ | .32^**^ | .44^**^ | .33^**^ | .37^**^ | .52^**^ | .38^**^ | .50^**^ | .36^**^ | .42^**^ | .42^**^ | .48^**^ | .39^**^ | 1 |  |  |  |  |  |  |  |  |
|  |  | 1411 | 1273 | 2005 | 1745 | 1761 | 1760 | 1045 | 1392 | 1371 | 1097 | 1225 | 1168 | 1165 | 1300 | 1513 | 2308 | 2070 | 2025 | 2476 | 2170 | 2375 | 354 | 2774 | 2802 |  |  |  |  |  |  |  |  |
| **25** | Maths GCSE 16  n | -.35^**^ | -.24^**^ | .52^**^ | .41^**^ | .30^**^ | .45^**^ | .57^**^ | .35^**^ | .46^**^ | .58^**^ | .52^**^ | .39^**^ | .37^**^ | .37^**^ | .61^**^ | .63^**^ | .48^**^ | .41^**^ | .49^**^ | .51^**^ | .36^**^ | .73^**^ | .44^**^ | .50^**^ | 1 |  |  |  |  |  |  |  |
|  |  | 2405 | 2124 | 3640 | 3418 | 3443 | 3492 | 1956 | 2438 | 2391 | 2075 | 2132 | 1989 | 1983 | 2283 | 2541 | 3701 | 3181 | 3088 | 4020 | 3366 | 3690 | 380 | 2511 | 2196 | 5707 |  |  |  |  |  |  |  |
| **26** | Maths Web 16  n | -.42^**^ | -.31^**^ | .50^**^ | .36^**^ | .28^**^ | .42^**^ | .57^**^ | .31^**^ | .43^**^ | .58^**^ | .56^**^ | .41^**^ | .39^**^ | .38^**^ | .57^**^ | .67^**^ | .48^**^ | .41^**^ | .48^**^ | .52^**^ | .39^**^ | .72^**^ | .45^**^ | .51^**^ | .75^**^ | 1 |  |  |  |  |  |  |
|  |  | 2489 | 2286 | 1741 | 2027 | 2039 | 2056 | 1449 | 1866 | 1835 | 1558 | 1737 | 1623 | 1617 | 1830 | 940 | 1877 | 1650 | 1614 | 2021 | 1742 | 1921 | 287 | 1456 | 1333 | 2203 | 2521 |  |  |  |  |  |  |
| **27** | Speed Proc. 16  n | .20^**^ | .27^**^ | -.24^**^ | -.15^**^ | -.14^**^ | -.21^**^ | -.26^**^ | -.14^**^ | -.20^**^ | -.24^**^ | -.20^**^ | -.13^**^ | -.15^**^ | -.17^**^ | -.24^**^ | -.28^**^ | -.19^**^ | -.21^**^ | -.26^**^ | -.22^**^ | -.18^**^ | -.26^**^ | -.20^**^ | -.21^**^ | -.32^**^ | -.34^**^ | 1 |  |  |  |  |  |
|  |  | 2381 | 2189 | 1669 | 1945 | 1956 | 1975 | 1395 | 1791 | 1761 | 1495 | 1672 | 1563 | 1558 | 1760 | 899 | 1805 | 1589 | 1555 | 1945 | 1678 | 1849 | 271 | 1402 | 1289 | 2113 | 2391 | 2412 |  |  |  |  |  |
| **28** | Memory 16  n | -.23^**^ | -.18^**^ | .26^**^ | .12^**^ | .15^**^ | .19^**^ | .28^**^ | .12^**^ | .26^**^ | .26^**^ | .22^**^ | .11^**^ | .23^**^ | .15^**^ | .24^**^ | .29^**^ | .19^**^ | .23^**^ | .18^**^ | .22^**^ | .22^**^ | .36^**^ | .18^**^ | .31^**^ | .33^**^ | .38^**^ | -.23^**^ | 1 |  |  |  |  |
|  |  | 2410 | 2208 | 1689 | 1966 | 1978 | 1995 | 1413 | 1821 | 1790 | 1516 | 1691 | 1586 | 1580 | 1780 | 911 | 1830 | 1611 | 1578 | 1970 | 1700 | 1879 | 279 | 1425 | 1310 | 2138 | 2419 | 2368 | 2445 |  |  |  |  |
| **29** | Verbal 16  n | -.17^**^ | -.16^**^ | .28^**^ | .36^**^ | .23^**^ | .37^**^ | .33^**^ | .33^**^ | .24^**^ | .33^**^ | .26^**^ | .37^**^ | .22^**^ | .38^**^ | .30^**^ | .39^**^ | .45^**^ | .27^**^ | .50^**^ | .43^**^ | .23^**^ | .42^**^ | .43^**^ | .33^**^ | .43^**^ | .42^**^ | -.19^**^ | .19^**^ | 1 |  |  |  |
|  |  | 2662 | 2389 | 1851 | 2154 | 2168 | 2186 | 1535 | 1973 | 1939 | 1644 | 1829 | 1704 | 1698 | 1938 | 985 | 1975 | 1725 | 1686 | 2134 | 1829 | 2025 | 306 | 1522 | 1383 | 2336 | 2471 | 2365 | 2393 | 2697 |  |  |  |
| **30** | Non verbal 16  n | -.31^**^ | -.26^**^ | .31^**^ | .26^**^ | .26^**^ | .23^**^ | .33^**^ | .20^**^ | .36^**^ | .40^**^ | .38^**^ | .29^**^ | .42^**^ | .30^**^ | .36^**^ | .50^**^ | .36^**^ | .48^**^ | .33^**^ | .41^**^ | .40^**^ | .46^**^ | .36^**^ | .60^**^ | .49^**^ | .57^**^ | -.25^**^ | .31^**^ | .35^**^ | 1 |  |  |
|  |  | 2417 | 2233 | 1696 | 1972 | 1987 | 2002 | 1416 | 1819 | 1789 | 1517 | 1700 | 1589 | 1583 | 1788 | 914 | 1834 | 1613 | 1580 | 1974 | 1701 | 1877 | 280 | 1426 | 1311 | 2145 | 2436 | 2365 | 2390 | 2405 | 2449 |  |  |
| **31** | Reading 16  n | -.25^**^ | -.17^**^ | .38^**^ | .38^**^ | .27^**^ | .54^**^ | .45^**^ | .32^**^ | .31^**^ | .43^**^ | .34^**^ | .38^**^ | .26^**^ | .45^**^ | .42^**^ | .46^**^ | .45^**^ | .28^**^ | .66^**^ | .49^**^ | .25^**^ | .52^**^ | .46^**^ | .33^**^ | .50^**^ | .48^**^ | -.32^**^ | .25^**^ | .47^**^ | .37^**^ | 1 |  |
|  |  | 2624 | 2409 | 1820 | 2126 | 2140 | 2158 | 1518 | 1961 | 1930 | 1627 | 1816 | 1692 | 1686 | 1917 | 985 | 1960 | 1710 | 1669 | 2113 | 1809 | 2008 | 304 | 1517 | 1385 | 2308 | 2503 | 2393 | 2424 | 2608 | 2433 | 2661 |  |
| **32** | Language 16  n | -.25^**^ | -.22^**^ | .33^**^ | .42^**^ | .26^**^ | .35^**^ | .39^**^ | .32^**^ | .33^**^ | .37^**^ | .35^**^ | .41^**^ | .31^**^ | .39^**^ | .40^**^ | .46^**^ | .48^**^ | .35^**^ | .50^**^ | .55^**^ | .26^**^ | .47^**^ | .48^**^ | .38^**^ | .49^**^ | .52^**^ | -.24^**^ | .24^**^ | .46^**^ | .43^**^ | .53^**^ | 1 |
|  |  | 2531 | 2321 | 1769 | 2065 | 2076 | 2093 | 1479 | 1904 | 1871 | 1584 | 1768 | 1651 | 1645 | 1863 | 951 | 1903 | 1672 | 1634 | 2050 | 1766 | 1947 | 292 | 1481 | 1352 | 2235 | 2501 | 2392 | 2422 | 2510 | 2430 | 2537 | 2563 |
| *Note*. n = number of participants. ** Significant at the 0.01 level (2-tailed). Smaller scores for Number line estimation, Dot estimation and Speed of processing index better performance, therefore the correlations among these 3 measures are positive among each other and negative with all other measures. | | | | | | | | | | | | | | | | | | | | | | | | | | | | | | | | | |
